# A critical revision of the fossil record, stratigraphy and diversity of the Neogene seal genus *Monotherium* (Carnivora, Phocidae)

**DOI:** 10.1098/rsos.171669

**Published:** 2018-05-09

**Authors:** Leonard Dewaele, Olivier Lambert, Stephen Louwye

**Affiliations:** 1 Department of Geology, Ghent University, Ghent, Belgium; 2 ‘Earth and History of Life’, Royal Belgian Institute of Natural Sciences, Brussels, Belgium

**Keywords:** Phocidae, *Monotherium*, Neogene, North Atlantic, North Sea Basin

## Abstract

Historically, *Monotherium* had been one of the few genera of extinct Phocidae (true seals) that served as a wastebin taxon. Consequently, it did neither aid in understanding phylogenetic relationships of extinct Phocidae, nor in understanding seal diversity in deep time. This urged the reassessment of the genus. Before our review, *Monotherium* included five different species: *Monotherium aberratum*, *Monotherium affine*, and *Monotherium delognii* from Belgium; *Monotherium gaudini* from Italy; and *Monotherium*? *wymani* from the east coast USA. In this work we redescribe the fossil record of the genus, retaining the type species *M. delognii*. *Monotherium aberratum* and *M. affine* are reassigned to the new phocine genus *Frisiphoca*. *Monotherium gaudini* is renamed and considered a stem-monachine (*Noriphoca gaudini*). The holotype of the monachine *M.*? *wymani* requires further study pending the discovery of new fossil material that could be attributed to the same taxon. Reinvestigating the stratigraphic context reveals that *N. gaudini* most likely represents one of the two oldest named phocid seals, or even the oldest, dated to the late Oligocene–earliest Miocene. Our results allow questioning the widespread idea that Phocidae originated in the western Atlantic and better appreciate their palaeobiogeography during the late Oligocene–Miocene interval in the North Atlantic realm.

## 1. Introduction

The extinct genus *Monotherium* Van Beneden, 1876 (*Monatherium* in Van Beneden, 1877) is a particular taxon among Phocidae (true seals). Of all currently known extant and extinct phocid seal taxa, most are considered monospecific or include only two species [1], and only two genera include three species: the extant *Pusa* and the extinct middle to late Miocene *Praepusa* [2,3]. However, the Miocene monachine genus *Monotherium* surpasses these and currently includes five species: *Monotherium aberratum* Van Beneden, 1876; *Monotherium affine* Van Beneden, 1876; *Monotherium delognii* Van Beneden, 1876; *Monotherium gaudini* (Guiscardi, 1870); and *Monotherium*? *wymani* (Leidy, 1853), from Belgium (first three species), Italy and the east coast of the United States, respectively. Furthermore, historically other species have been assigned to this genus, e.g. *Monotherium maeotica* [*Cryptophoca maeotica*] (Nordmann, 1860), and *Monotherium rugosidens* (Owen, in Adams, 1879) [holotype is odontocete tooth] [4,5]. On the other side, *M. aberratum*, *M. affine* and *M. delognii* have been recently considered nomina dubia [6], but without any evidence provided. A major issue for the taxonomy of *Monotherium* is that the fossil record of the different species within the genus varies strongly in terms of preserved skeletal elements: most *Monotherium* species cannot be directly compared to other species within the genus. For example, *M. gaudini* is known from a partial skull [7], while no cranial bones have been attributed to the other species of the genus.

In the light of recently discovered specimens showing affinities with part of the species of this genus, a reinvestigation is essential, with a description of the new material and reassessment of the previously described species.

## 2. Material and methods

### 2.1. Terminology and comparative material

This study follows the anatomical nomenclature of the recent publications of Amson & Muizon [8], Berta *et al*. [6] and Dewaele *et al*. [9,10]. Whenever terms have not been used in any of the aforementioned publications, anatomical nomenclature follows Evans & Lahunta's description of the dog [11]. Length measurements were taken to the nearest 0.1 mm, using analogue calipers and are presented as tables 2, 4 and 5 and electronic supplementary material (Supplementary Information 1: tables S1–S3). For reasons of consistency, these measurements were taken following the same scheme as Koretsky [2], which has more recently been applied to other extinct phocids [6,9,10]. Comparative specimens of extant and extinct taxa are listed as electronic supplementary material (Supplementary Information 1: lists 1 (extant taxa) and 2 (extinct taxa)).

**Table 1.** Selection of cranial measurements of *Noriphoca gaudini* redrawn from Guiscardi [7] (originally for *Phoca gaudini*).

	length (mm)	
bizygomatic width skull	184.0	
sagittal length of the palate	122.0	
transverse width across pterygoid processes	63.0	
width across the canines (excluding canines)	36.0	
width across the canines (including canines)	61.0	
width across the incisor tooth row	29.0	
length of postcanine tooth row	83.0	
	anteroposterior length (mm)	labiolingual length (mm)
diameter upper premolars		
first premolar	11.2	7.6
second premolar	17.4	9.0
third premolar	16.8	9.5
diameter upper first molar (isolated specimen)	12.0	7.0

**Table 2.** Measurements of the humerus of *Frisiphoca aberratum* and *Frisiphoca affine* (in mm). Measurements based on the scheme presented by Koretsky [2].

	*Frisiphoca aberratum*	*Frisiphoca affine*
	IRSNB 1191-M266 (lectotype)	IRSNB 1118-M260 (lectotype)
total length	142.5	177.1
length deltopectoral crest	77.0	n.a.
height head	33.9	36.8
height trochlea	n.a.	2.28
width head	35.9	41.5
width proximal epiphysis	48.3	60.2
width distal epiphysis	53.4	61.8
distal width trochlea	32.8	n.a.
transverse width mid-diaphysis	22.0	24.8

**Table 3.** Measurement of the robustness of phocid humeri, adapted from Muizon & Bond [71]. The robustness *R* is calculated as the ratio of *l*/*L* or (*l*_1_ + *l*_2_ + *l*_3_ + *l*_4_)/*L*, with *l*_1_ = maximum transverse width of the proximal epiphysis, *l*_2_ = minimum transverse width of the diaphysis, *l*_3_ = maximum transverse width of the distal epiphysis, *l*_4_ = maximum anteroposterior width of the diaphysis at the level of the deltoid tuberosity; *L* = maximum length of the humerus. Measurements with an asterisk are estimations. Measurements in mm. Sources provided for measurements retrieved from the literature. Measurements by Koretsky [2] represent averages of multiple specimens.

taxon	*l*_1_	*l*_2_	*l*_3_	*l*_4_	*l*	*L*	*R* = *l*/*L*
*Acrophoca longirostris* [71]	65.0	26.2	53.0	57.5	201.7	154	1.309
	64.6	26.4	54.8	51.0	201.5	155	1.300
	62.0	27.6	55.5	51.2	196.3	153	1.283
	63.0	28.0	53.2	51.0	195.2	144.6	1.349
	62.0	26.2	51.7	52.5	192.4	145.7	1.320
	61.0	30.0	51.0	54.0	196.0	146.0	1.340
*Cryptophoca maeotica* [2]	34.2	14.5	37.0	33.5	119.2	107.1	1.113
*Halichoerus grypus*	50.9	25.3	52.7	40.9	169.8	124.4	1.365
	56.8	24.2	58.4	41.2	180.6	134.5	1.343
*Homiphoca capensis* [71]	62.8	25.0	51.5	53.5	192.8	136.4	1.413
	55.1	20.7	45.7	46.5	168.0	119.4	1.407
*Hydrurga leptonyx* [71]	75.3	36.6	64.3	76.5	252.7	166.0	1.522
	78.0	34.0	67.0	90.0	269.0	168.0	1.601
*Leptonychotes weddellii* [71]	59.7	26.0	58.3	58.7	202.7	139.7	1.450
	66.0	26.0	59.0	65.0	216.0	154.0	1.402
*Leptophoca proxima*	38.0	14.4	38.0	37.3	127.7	131.7	0.970
*Lobodon carcinophaga* [71]	67.4	27.0	57.0	63.5	214.9	132.3	1.624
	65.0	32.7	62.0	69.7	227.5	124.0	1.826
	59.0	29.0	55.0	59.0	202.0	119.0	1.697
*Mirounga leonina* [71]	130.0	60.0	130.0	133.0	453.0	290.0	1.562
*Monachus monachus* [71]	61.0	26.7	58.4	56.0	202.1	144.0	1.403
*Nanophoca vitulinoides* [9]	27.5	9.8	24.0	20.0	81.3	72.4	1.123
	28.1	9.5	26.6	20.8	85.0	78.2	1.087
*Phoca vitulina*	48.5	18.0	41.5	36.2	144.2	110.4	1.306
	49.9	18.8	44.2	36.4	149.3	122.9	1.215
*Phocanella pumila*	45.7	15.8	47.1	39.9	148.5	127.8	1.162
*Piscophoca pacifica* [71]	64.1	28.0	55.5	63.2	211.2	148.9	1.418
	64.5	30.3	62.0	63.6	220.4	160.8	1.370
*Praepusa vindobonensis* [2]	27.6	10.6	25.6	24.2	88.0	86.3	1.020
*Properiptychus argentinus* [71]	58.8	18.4	40.7	40.0*	157.9	125.9	1.254
	46.0	18.0	42.0	40.0	146	120.9	1.207
*Pusa sibirica*	36.6	12.6	36.1	24.8	110.1	91.4	1.205
	43.1	15.4	42.4	32.7	133.6	103.1	1.296
*Frisiphoca aberratum*	48.3	22.0	53.4	51.0*	174.7	142.5	1.226

**Table 4.** Measurements of the astragalus IRSNB 1126-M262, identified as Phocidae aff. *Frisiphoca affine* (in mm). ‘+’ indicates that the measured length is smaller than the real length, due to post-mortem wear of the specimen.

	Phocidae aff. *Frisiphoca affine*
	IRSNB 1126-M262
absolute length	+75.5
maximum dorsoplantar height	+47.7
mediolateral width across tibial facet	+31.5
dorsoplantar height astragalar head	+28.0
mediolateral width astragalar head	n.a.
dorsoplantar height caudal process	+30.0
mediolateral width caudal process	+16.6
maximal length ectal facet	n.a.
maximal length sustentacular facet	+22.7

**Table 5.** Measurements of the calcaneum IRSNB 1125-M263 identified as Phocidae aff. *Frisiphoca affine* (in mm).

	Phocidae aff. *Frisiphoca affine*
	IRSNB 1125-M263
absolute proximodistal length	78.2
maximal mediolateral width	n.a.
least mediolateral width of calcaneal tuber	n.a.
mediolateral width across the medial calcaneal tuberosity	n.a.
maximal dorsoplantar height	38.8
maximal length of ectal facet	20.8
height of ectal facet	8.6
maximal length of sustentacular facet	33.6
mediolateral width of facet for navicular	n.a.
dorsoplantar height of facet for navicular	24.9

### 2.2. Dinoflagellate cyst biostratigraphy

The palynological preparation of the sediments followed standard techniques described by Louwye *et al*. [12]. Acid treatments with HCl and HF were applied for the removal of carbonates and silicates, respectively. Sieving of the organic residue was carried out on a nylon screen with a 10 µm mesh size. The residue was placed on glass slides with glycerol gelatin jelly. The microscopic analysis was carried out with a transmitted light microscope Zeiss AxioImager A1 under 400× magnification. The entire slide was scanned in non-overlapping traverses. The taxonomy of the dinocysts and acritarchs follows Fensome *et al*. [13]. A table showing all the observed dinocyst and acritarch taxa is presented as electronic supplementary material (Supplementary Information 1: table S4).

### 2.3. Phylogenetic analysis

The phylogenetic analysis largely follows the methodology of Dewaele *et al*. [9] for the assessment of the phylogenetic position of *Nanophoca vitulinoides* Dewaele, Amson, Lambert & Louwye, 2017 among Phocidae. The analysis was performed using PAUP version 4.0b10 for Macintosh [14] with a heuristic search option with simple sequence addition, using the tree-bisection-reconnection (TBR) algorithm. Bootstrap values were obtained after a full heuristic search with 10 000 replications with random number seed zero and the best tree saved for each replication. Character states were optimized with accelerated transformation criterion (ACCTRAN). The phylogenetic analysis has been performed both without down-weighting homoplastic characters and with the *k*-value of the Goloboff criterion set at three, for down-weighting homoplastic characters. The phylogenetic matrix includes 80 morphological characters (Supplementary Information 1: list 3, table S5; Supplementary Information 2) and 27 operational taxonomic units (OTUs), including the extinct Pinnipedimorpha *Enaliarctos mealsi* Mitchell & Tedford, 1973 and *Pteronarctos goedertae* Barnes, 1989, the Otariidae *Otaria byronia* Blainville, 1820 (extant) and *Thalassoleon mexicanus* Repenning & Tedford, 1977 (extinct), and the desmatophocid *Allodesmus kernensis* Kellogg, 1922 as outgroup taxa; the extinct Monachinae *Acrophoca longirostris* Muizon, 1981, *Hadrokirus martini* Amson & Muizon, 2013, *Homiphoca capensis* (Hendey & Repenning, 1971), *Piscophoca pacifica* Muizon, 1981, and *Pliophoca etrusca* Tavani, 1941; the extant Monachinae *Hydrurga leptonyx* (Blainville, 1820), *Leptonychotes weddellii* (Lesson, 1826), *Lobodon carcinophaga* (Hombron & Jacquinot, 1842), *Mirounga leonina* (Linnaeus, 1758), *Monachus monachus* Hermann, 1779, and *Ommatophoca rossii* (Gray, 1844); the extinct Phocinae *Devinophoca claytoni* Koretsky & Holec, 2002, *Kawas benegasorum* Cozzuol, 2001, *Leptophoca proxima* (Van Beneden, 1876), *Nanophoca vitulinoides* (Van Beneden, 1871); and the extant Phocinae *Erignathus barbatus* Erxleben, 1777, *Halichoerus grypus* (Fabricius, 1791), and *Phoca vitulina* Linnaeus, 1758. Four (former) *Monotherium* species are included for the first time in a phylogenetic analysis: *Monotherium aberratum* (Van Beneden, 1876; as *Frisiphoca aberratum*), *Monotherium affine* (Van Beneden, 1876; as *Frisiphoca affine*), *Monotherium gaudini* (Guiscardi, 1870; as *Noriphoca gaudini*) and *Monotherium*? *wymani* (Leidy, 1853)*.* Two characters are parsimony-uninformative (23, 35) and three (31, 35, 76) are ordered. The choice of outgroups is such that early stem Pinnipedimorpha are represented (*E. mealsi* and *Pt. goedertae*), as well as two of the three non-phocid pinniped families with Desmatophocidae (*A. kernensis*) and Otariidae (*O. byronia* and *T. mexicanus*). Odobenidae are excluded in order to keep the outgroup appreciably small.

### 2.4. Institutional abbreviations

ELNRP, East Libya Neogene Research Project collection, housed at Garyounis University, Benghazi, Libya; IRSNB, Institut Royal des Sciences Naturelles de Belgique (‘M’ representing type and figured specimens from the fossil mammal collection), Brussels, Belgium; MCZ, Museum of Comparative Zoology, Harvard University, Cambridge, Massachusetts, USA; MSNUN, Museo di Storia Naturale del'Università di Napoli, Naples, Italy; USNM, Department of Paleobiology, National Museum of Natural History, Washington, DC, USA.

## 3. Historical background

Prior to the current study, five species have been considered within the genus *Monotherium*: *Monotherium aberratum* Van Beneden, 1876; *Monotherium affine* Van Beneden, 1876; *Monotherium delognii* Van Beneden, 1876; *Monotherium gaudini* (Guiscardi, 1870); and *Monotherium*? *wymani* (Leidy, 1853). Unfortunately, as with many historically longstanding extinct taxa, its history has been turbulent. *Monotherium*? *wymani* and *M. gaudini* have been described prior to the erection of the genus *Monotherium*, but had been named *Phoca wymani* Leidy, 1853, on the basis of a few isolated cranial and postcranial specimens from the Miocene (presumably the Calvert formation) of Richmond, Virginia [15], and *Phoca gaudini* Guiscardi, 1870, on the basis of one partial skull and a mandible from 3 km east of Roccamorice, Abruzzo Region, Italy [7], respectively. Later, the genus *Monotherium* was erected by Van Beneden [16], including *M. delognii*, *M. affine* and *M. aberratum* based on isolated postcranial material from Antwerp (Belgium, southern margin of the North Sea Basin). Although somewhat vague and very concise, Van Beneden [16] provided descriptive elements for *M. delognii* (‘similarities with *Phoca barbata*’) and *M. aberratum* (‘size greater than *Monachus*’), but not for *M. affine*. However, it is argued that *M. affine* comprises all material from the original collection that could not be assigned to either of the two other species. For neither of the three species, Van Beneden [16] provided illustrations or collection numbers.

Having met the requirements of naming a species under the International Code on Zoological Nomenclature (ICZN) Article 11, and being published before 1931, ICZN Article 12 applies to *Monotherium* and ‘indications’ suffice for naming taxa. Therefore, *Monotherium* is a valid name, irrespective of Van Beneden [17] using the name *Monatherium* one year later and implicitly providing the etymology of *Monatherium*, and rendering the original name of *Monotherium* a typographical error*.* Van Beneden corrected the spelling, providing the etymology, after observing affinities between the genus ‘*Monatherium*’ and the extant *Pelagius monachus*, which is a junior synonym of *Monachus monachus*. Therefore, it can safely be implied that the name *Monatherium* etymologically refers to *Monachus monachus* and that the former name of *Monotherium* by Van Beneden [16] is a typographical error; yet, *Monotherium* is the valid name for the taxon and *Monatherium* should be considered a junior synonym of it. In the 1877 publication, Van Beneden [17] described *Monotherium delognii*, *Monotherium affine* and *Monotherium aberratum* in much more detail and provided the collection numbers of specific specimens.

Van Beneden did not assign a type species to the genus *Monotherium*. In 1922, Kellogg retained the name *Monotherium* for all three taxa and assigned *Monotherium delognii* as the type species of the genus on the basis of page priority in Van Beneden [17] [18, p. 72]. He also regarded *Monotherium affine* as a junior synonym to *M. delognii*, stating ‘*Monotherium delognii* is based upon too fragmentary material to distinguish it from *Monotherium affine*. Therefore, since *Monotherium delognii* has page priority, it is here interpreted to include Van Beneden's second species, *Monotherium affine*, as well.’ Kellogg [18] also retained *Monotherium affine* as a separate taxon and renamed the Italian species *Phoca gaudini* to *Monotherium gaudini*.

More recently, Ray [15] studied the North American *Phoca wymani* in detail, removing it from the genus *Phoca* and tentatively placing it among *Monotherium*: *Monotherium*? *wymani*. However, *M*.? *wymani* is based on very fragmentary isolated specimens that bear little diagnostic value for comparison with other extinct Phocidae. Following Kellogg [18], Ray [15] did not use the generic name *Monatherium*, but instead used the first, and correct, name *Monotherium* for the three taxa described by Van Beneden [16,17].

## 4. Dinoflagellate cyst biostratigraphy of *Monotherium* from Belgium

Historically, the stratigraphic context of *Monotherium aberratum*, *Monotherium affine* and *Monotherium delognii* had been poorly defined. Van Beneden [17] assigned a ‘Diestian’ age to the entire record of *Monotherium* from Belgium. However, the ‘Diestian’ is a currently abandoned term and it had been shown that the term should not be used any more [19]. Moreover, shortly after the description of *Monotherum* by Van Beneden [16,17], the ‘Anversien’ (=‘Antwerpian’) was erected and considerably restricted the extent of the ‘Diestian’ [19,20]. Nowadays, the same toponym is used for the upper Miocene Diest Formation, which is roughly the lithostratigraphical equivalent of the ‘Diestian’ stage. The Diest Formation is a diachronous formation deposited in a marginal marine setting [19]. Near the city of Antwerp, the deposits of the Diest Formation (Deurne Member) are of late Tortonian age [19–23].

Only two sediment samples could be recovered from bone cavities of specimens formerly attributed to the genus *Monotherium*: sample 1108LDW-1100Lab from the thoracic or lumbar vertebrae of *Monotherium delognii* (either from specimen IRSNB 1108 or from specimen IRSNB 1108-M255; Phocidae indet. in this study, see Supplementary Information 3) and sample 1132LDW-1102Lab from the cervical or thoracic vertebrae originally assigned to *Monotherium aberratum* (IRSNB 1132-M269; Phocidae indet. in this study, see Supplementary Information 3). The samples were palynologically analysed for organic-walled dinoflagellate cysts (dinocysts) and acritarchs (see Supplementary Information 1, table S4).

The preservation and diversity of the dinocysts in sample LDW1108-1100Lab is poor. A total of 10 dinocyst species and one reworked acritarch were recorded (Supplementary Information 1: table S4). Dybkjaer & Piasecki [24] defined the *Achomosphaera andalousiensis* zone as the interval from the lowest common occurrence of the eponymous species to the lowest occurrence of *Gramocysta verricula*, and suggest an age of 13.2 Ma for the lower boundary of the zone. The dinoflagelatte cysts thus indicate a maximum age of 13.2 Ma (early Serravallian, late middle Miocene) for the sediment sample. The other recorded dinoflagellate cysts are stratigraphically long ranging species with no biostratigraphical value.

The preservation and diversity of the dinocysts in sample LDW1132-1102Lab is similarly poor. Only eleven dinocyst species and two acritarch were recorded. A maximum age for the sample is provided by the key species *Habibacysta tectata.* This species has a lowest occurrence in high latitudes dated at 14.2 Ma by Schreck *et al*. [25], and this datum was later confirmed by Quaijtaal *et al*. [26] in lower latitudes (Porcupine Basin, off southwest Ireland). A lowest occurrence of *Operculodinium*? *eirikianum* at *ca* 14 Ma (upper Langhian) is provided by Louwye *et al*. [27] in a low-resolution palynological study of the Miocene of the Porcupine Basin. *Operculodinium*? *eirikianum* has a persistent highest occurrence at the lower–upper Pliocene boundary at *ca* 2.617 Ma [28]. The lowest occurrence of *Operculidinium tegillatum* is located at the Tortonian–Messinian boundary (7.25 Ma). The persistent highest occurrence is noted in the Zanclean at 3.7 Ma [28]. *Quinquecuspis concreta* has a poorly specified lowest occurrence in the upper Tortonian of Germany [29]. The dinoflagellate cysts in this second sample indicate an age situated between *ca* 7.25 Ma (or somewhat older in the late Tortonian), and 3.7 Ma (late Zanclean).

## 5. Systematic palaeontology

**Family Phocidae Gray, 1821**

**Subfamily Monachinae Gray, 1869**

**Genus *Noriphoca* gen. nov**.

*LSID*. urn:lsid:zoobank.org:act:CF6ABB16-8EEA-4490-8D6D-918B48910613

*Type and only included species*. *Noriphoca gaudini* (Guiscardi, 1870).

*Diagnosis*. As for the only included species

*Etymology*. From the Greek adjective ‘*noris*’ and the Greek noun ‘*phoke*’. Meaning ‘early’ and ‘seal’, respectively, referring to the geologically old age of the species. An age interval of late Oligocene to early Miocene is presented here (see below). Hence, this taxon may possibly represent the first unquestionable phocid from the Palaeogene ([10] versus [30,31]).

***Noriphoca gaudini* (Guiscardi, 1870)**

(figures 1 and 2)

**Figure 1.**
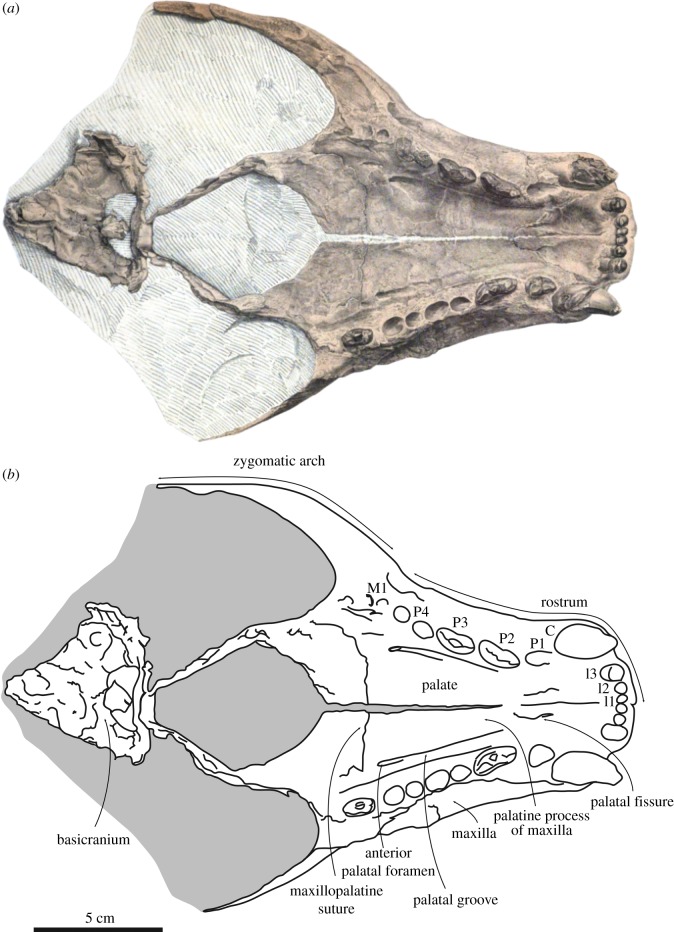
Holotype skull of the stem monachine *Noriphoca gaudini*, MSNUN123, presumably from the late Oligocene–early Miocene *Lepidocyclina* Limestone of the Bolognano Formation near Roccamorice, Italy, and originally described as *Phoca gaudini* by Guiscardi ([7]: plate 1). Original drawing from Guiscardi [7] (*a*), and line drawing (*b*). Skull in ventral view. Sediment and obliterated parts are indicated in grey. Scale bar equals 5 cm.

**Figure 2.**
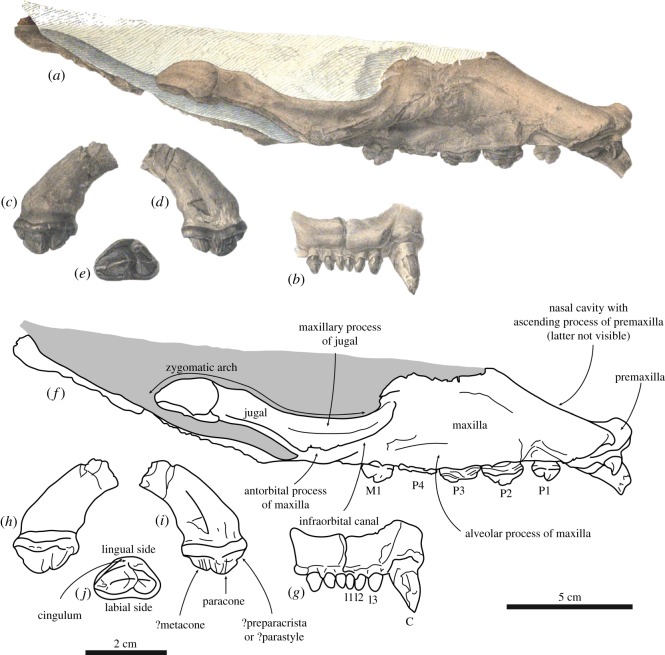
Holotype skull of the stem monachine *Noriphoca gaudini*, MSNUN123, presumably from the late Oligocene–early Miocene *Lepidocyclina* Limestone of the Bolognano Formation near Roccamorice, Italy, and originally described as *Phoca gaudini* by Guiscardi ([7]: plate 2), also including isolated teeth originally assigned to *P. gaudini*. Original drawing from Guiscardi [7] (*a*–*e*), and line drawing (*f*–*j*). Skull in right lateral view (*a*,*f*), and snout in anterior view (*b*,*g*). Corresponding scale bar equals 5 cm. Isolated right postcanine tooth in lingual (*c*,*h*), labial (*d*,*i*) and occlusal (*e*,*j*) view. Corresponding scale bar equals 2 cm. Sediment and obliterated parts are indicated in grey.

*LSID*. urn:lsid:zoobank.org:act:0A15AA71-8B86-495B-BF9B-5FE3D11FB2CF

*Diagnosis*. Large phocid, comparable in size to the leopard seal, *Hydrurga leptonyx*. Stem phocid, but still yielding typically monachine characters in having: an ascending process of the premaxilla that is (at least partially) within the nasal cavity and not visible laterally; roots of incisors not (or only weakly) laterally compressed. The anterior termination of the maxillary process of the jugal is located lateral to the infraorbital foramen, which is shared with extinct Monachinae (*Acrophoca longirostris*, *Hadrokirus martini*, *Homiphoca capensis* and *Piscophoca pacifica*) and the extant Monachinae *Mirounga*. In the phylogenetic analysis, the identification of *Noriphoca gaudini* as a separate taxon is supported by one unequivocal autapomorphy: the ventral edge of the zygomatic arch is level with the alveolar plane. Furthermore, the skull of *N. gaudini* differs from all other Monachinae by the presence of three upper incisors, and a paracone on the postcanine teeth that is low.

*Holotype*. MSNUN123, partial skull. Only the ventral and anterior portion of the skull are visible. The dorsal portion of the skull is missing and the preserved part is embedded in the matrix, inhibiting description of the specimen in dorsal view.

*Type locality*. Approximately 3 km east of the village of Roccamorice (Abruzzo Region, Italy) [7].

*Stratigraphy and age*. Guiscardi [7] noted that the specimen comes from calcareous deposits, rich in bitumen. Based on more recent literature, it is evident that the outcropping formations, 3 km east of Roccamorice are the Santo Spirito and Bolognano formations [32,33]. Because it had been hypothesized that the earliest Phocidae lived around 23 Ma (divergence date of Phocidae from other Pinnipedia taken from Higdon *et al*. [34]), around the Oligocene–Miocene boundary, the late Oligocene to Miocene Bolognano Formation is the most likely candidate as the origin of the *Monotherium gaudini* holotype. It is indeed very unlikely that the holotype of *M. gaudini* comes from the underlying Eocene Santo Spirito Formation, which also outcrops in the area. Furthermore, the bituminous layers of the Bolognano Formation are found in its lower part, mainly restricted to the *Lepidocyclina* Limestone dated to the Oligocene or earliest Miocene (Aquitanian) ([33,35]; and references therein). Consequently, the exact age of the holotype specimen of *M. gaudini* is still unknown; but nevertheless, this holotype is most likely as old as, or even older than the Aquitanian. Hence, *N. gaudini* is most likely older than *Afrophoca libyca*, known from the Burdigalian of Libya [36].

*Remarks*. Other specimens from the same locality and from the same level, i.e. a partial mandible and isolated teeth, had been presented by Guiscardi [7], but these specimens are currently lost (Giovanni Bianucci 2017, personal communication). Additionally, the little informative illustration of the mandible ([7]: fig. 6) precludes its description. Therefore, we do not deem it appropriate to redescribe these specimens in depth. However, the isolated teeth are comparable in shape to the teeth of the holotype skull and are unspecialized (contrasting to extant Monachinae, except *Monachus*). In their review of the palaeobiogeography of Pinnipedia, Deméré *et al.* [4] disregarded *Monotherium gaudini*, but referred to cf. *Monotherium* sp. indet. isolated teeth from the Bismantova Formation in the Stirone River, Italy, described by Cigala-Fulgosi & Pilleri [37] and Pilleri & Cigala-Fulgosi [38]. As noted by Dewaele *et al*. [10], the geological age of the Bismantova Formation is strongly debated, with proposed ages ranging from the Burdigalian–early Langhian [39] (adopted by Berta *et al*. [6]) to the late Langhian–Serravalian–early Tortonian [40,41] (adopted by Deméré *et al*. [4]). Although these teeth are clearly monachine, we observe only few similarities between those and the teeth of the holotype of *Noriphoca gaudini*. Although both have strongly pronounced cusps and a crenulated enamel layer, the cusps of the teeth of *N. gaudini* are much less raised than in the specimens from the Stirone River. Therefore, we deem it impossible to identify these isolated teeth more precisely than Monachinae indet. and they will no further be considered in this study. The redescription of *M. gaudini* (as *N. gaudini*) is entirely based on observations made on the descriptions, images and drawings presented by Guiscardi [7]. We, the authors, have not studied the type specimen in person.

Description and comparison

The type and only known specimen of *Noriphoca gaudini* comprises an incomplete skull (figures 1 and 2). The known parts of the skull include the ventral part of the rostrum and parts of the basicranium and the right zygomatic arch. A number of maxillary teeth are known as well [7]. The dorsal portion of the skull is missing and only the anterior portion of the snout is visible in dorsal view. The remainder of the skull is embedded in the matrix.

Only the anterior portion of the premaxilla is preserved and it is restricted to the interior of the nasal cavity, which means that it is not visible in lateral view. This is a typically monachine characteristic [42,43]. The anterior alveolar plane faces anteroventrally and the canines have a strong anterior aspect in their orientation. Amson & Muizon [8] observed a similar condition in the monachine *Homiphoca capensis*, but we conclude that this is considerably more pronounced in *N. gaudini* than it is in *H. capensis*. In lateral outline, the nasal cavity is weakly curved and almost rectilinear, and has a strong dorsal aspect to its orientation. This corresponds, notably, with *Acrophoca longirostris* from the late Miocene of Peru, and *Allodesmus* spp. [42,44–46]. Other Monachinae and pinnipedimorphs usually have snouts that face more anteriorly and that are either more strongly concave in lateral view (Phocidae), or convex (early pinnipedimorphs), or vary between concave or convex in lateral view within a single clade [8,42,47–57].

The palate of *N. gaudini* is slightly constricted at the level of the first premolar, after which the combined tooth rows diverge posteriorly. This is a typically phocid characteristic [58] and it is far less expressed in stem pinnipedimorphs and other non-phocid pinnipeds, where this constriction is minimal or absent [8,44–55,58]. The posterior divergence of the tooth row is minimal in early pinnipedimorphs, Odobenidae and Otariidae [47–55], but is present to varying degrees in Desmatophocidae and Phocidae [8,42,44–46]. On the palate, a small, slit-like and narrow palatine fissure is located at the suture between the premaxilla and maxilla, at the level of P1. The shape of the palatine fissure varies among extinct Phocidae and is, for instance, small in *Homiphoca capensis* [56,57], but large in *Hadrokirus martini* and *Piscophoca pacifica* [8,42], and this palatine fissure is generally large in other extinct Pinnipedia and early pinnipedimorphs [47–55], although a strongly reduced palatine fissure has also been observed in some desmatophocids [44]. A significant portion of the maxilla is preserved. The alveolar process, bearing the teeth, is slightly raised over the palatine process of the maxilla. In lateral view, the alveolar process faces ventrally, as in other Pinnipedimorpha, except the Monachinae *Hadrokirus martini*, *Ommatophoca rossii* and *Piscophoca pacifica* [8]. The palate is slightly arching dorsally in *N. gaudini*. This condition varies within different clades of Pinnipedimorpha. Among stem pinnipedimorphs, for instance, it is arching in *Enaliarctos* spp., but nearly flat in *Pinnarctidion bishopi* [47]. Even among Monachinae, Muizon [42] noted variation in the degree of arching of the palate. The palatal groove on the palatine process of the maxilla is narrow and becomes gradually more pronounced towards the anterior palatal foramen, which is located at the level of the posterior foramen of P4. Among Phocidae, the location of the anterior palatal foramen varies from the level of P3 (*Erignathus barbatus*, *Monachus* spp.) to posterior to the level of M1 (*Homiphoca capensis*, *Mirounga* spp., *Ommatophoca rossii* and *Pusa* spp.) [56,57,59]. Among extant and other Pinnipedimorpha, the position of this foramen varies, but it is generally well anterior to the level of the last postcanine tooth, notwithstanding exceptions such as *Desmatophoca brachycephala* [46–51]. The maxillopalatine suture is transversely straight between the M1. Consequently, for *N. gaudini*, the maxillopalatine suture is located posterior to the anterior palatine foramina. Muizon [42] noted that in some Phocinae, the anterior palatine foramina are located on the maxillopalatine suture, while they are anterior to that suture in Monachinae. Differences in the terminology used combined with inadequate illustrations inhibit studying this trait in detail for other pinnipedimorphs, based on literature alone. The posterior margin of the joined palatines is rounded, forming a half circle in *N. gaudini*. At the posterior extremity of the palatine, where right and left palatines meet, there is no true apex. This condition varies among Phocidae, ranging from a strongly-developed anterior invagination between the left and right palatines, to a caudal nasal spine of the palatines. Among other Pinnipedimorpha, the posterior margin of the palatines is smoothly rounded [44–54]. Compared to all other pinnipedimorphs, including Phocidae (except the Monachinae *Acrophoca longirostris* and *Hadrokirus martini*), the (rounded) posterior margin of the palatines is located relatively anterior, with the anteriormost tip located little posterior to the last postcanine tooth and the anterior extremity of the orbit in ventral view. In all other pinnipedimorphs, the palatine extends much more posteriorly, reaching the anteroposterior level of the jugal-squamosal contact and much more posterior to the last postcanine tooth.

The anterior margin of the infraorbital canal on the antorbital process of the maxilla is located at the level of M1. In Monachinae, this compares to the extant *Lobodon carcinophaga* and *Monachus* spp., and the extinct *Hadrokirus martini* and *Pi. pacifica*, whereas this canal is located either anterior (e.g. *Leptonychotes weddellii*) or posterior (e.g. to M1) in other Monachinae. In Phocinae, the anterior margin of the infraorbital canal is located posterior to M1. In stem pinnipedimorphs, the anterior margin of the infraorbital canal is usually located at the level of M1 [46–49]. Only rarely is the anterior margin of the infraorbital canal located anterior to M1 (e.g. *Enaliarctos* spp.) [52,53]. Related to that, the last postcanine is as the level of the root of the jugal process of the maxilla in *Noriphoca gaudini*, as in many Monachinae. In Phocinae, the last postcanine tooth is located anterior to the jugal process. In other early pinnipedimorphs and early pinnipeds, the last postcanine tooth reaches the level of the posterior portion of the root of the jugal process of the maxilla or posterior. The maxillary process of the jugal contacts the maxilla, terminating dorsal to the infraorbital canal, as in extinct Monachinae and *Mirounga*. In extant Monachinae (except *Mirounga*) and Phocinae, this process terminates lateral to the infraorbital canal. The jugal is incomplete, but comparison with more complete monachine skulls show that the anterior portion of the arch of the jugal is flat to slightly oriented downwards in *N. gaudini*. This condition varies among Monachinae: flat in *Acrophoca longirostris*, *Hadrokirus martini*, and *Mirounga*, upward in *Homiphoca capensis*, and *Piscophoca pacifica*, and downward in other Monachinae. In Phocinae, the anterior portion of the arch of the jugal is directed flat to upward.

In the upper tooth row, *Noriphoca gaudini* is characterized by having three incisors. Among Phocidae, the presence of three upper incisors is generally considered a characteristic of Phocinae (except *Cystophora cristata* having two upper incisors), while Monachinae are characterized by having two upper incisors. Three upper incisors are also present in early pinnipedimorphs and desmatophocids [18,44–49,53,54]. The lateral incisor I3 is larger than I1 and I2, which are similar in size, but still much smaller than the canine, and all incisors form a transversely straight row. In most Phocinae (except *Halichoerus grypus*) and early pinnipedimorphs, the lateral incisor is comparable in size to or only slightly larger than the medial incisor(s). A number of extinct Monachinae retain relatively small lateral incisors (*Hadrokirus martini*, *Homiphoca capensis* and *Piscophoca pacifica*), while the lateral incisor is clearly intermediate in size between medial incisors and canines in other extinct (*Acrophoca longirostris*) and extant Monachinae. Though, overall the incisors of *N. gaudini* are relatively smaller than the incisors in Monachinae (except *Acrophoca longirostris* and *Ommatophoca rossii*), while the incisors are almost always comparatively small in Phocinae (except *H. grypus*). The roots of the incisors of *N. gaudini* are not transversely compressed. In Monachinae and stem pinnipedimorphs, roots are not or only faintly compressed transversely, while in Phocinae, incisor roots are strongly compressed. Morphologically, there is little variation between the mesial incisors and the lateral incisors, apart from the size. The mesial incisors are more slender transversely than the lateral incisors. The incisors are single cusped and bear no cingulum. They are conical, but slightly recurved lingually and bear two occlusal facets on their lingual surfaces: one posterolateral and one posteromedial. Both canines are only partially preserved, but they are conical and appear labially curved. The canine alveolus is oval, i.e. slightly mediolaterally compressed.

The maxillary postcanine teeth include four premolars and one—noticeably smaller—molar. This is typically phocid, as other early stem pinnipedimorphs, desmatophocids, and many extinct odobenids and otariids have at least two upper molars [44–55,58]. Apart from the single-rooted P1, the postcanine teeth of *Noriphoca gaudini* are all double-rooted. This is common among Phocidae, in which only few taxa (*Halichoerus grypus* and *Mirounga* spp.) show a tendency towards single-rooted postcanine teeth. Whereas *Devinophoca claytoni* is the only known phocid to have a triple-rooted upper first molar [60], early stem pinnipedimorphs have triple-rooted upper first molars [46–49,52–54]. Desmatophocids, odobenids and otariids all show a trend towards reduction of the roots to single-rooted postcanine teeth during their evolution [44–46,50,58]. All postcanine teeth of *N. gaudini* are labiolingually broad and the lingual cingulum is well developed, yielding a semicircular or subtriangular shape in occlusal view. This is clearly a plesiomorphic character observed in early stem pinnipedimorphs, *Hadrokirus martini*, *Monachus* spp., and *Piscophoca pacifica*, while other (extant) Phocidae all display a specialized dentition with the cingulum being strongly reduced or absent [8,46–49,53,54].

The first premolar (P1) is subtriangular in occlusal view and is implanted parallel to the tooth row. The labial margin is slightly concave, while the lingual margin strongly projects lingually, yielding a subtriangular outline in occlusal view. The paracone (central cusp) and the ?metacone distal to the paracone are lowly raised and only little defined. The ?metacone is closely appressed against the paracone, further reducing the prominence of both. The term ‘metacone’ is based on usage in publications on other pinnipedimorphs [47], but contrasts with dedicated literature on dentition in carnivorans. For example, Solé *et al*. [61] stated that there is no metacone present on the maxillary premolars of *Dormaalocyon latouri*, the oldest known carnivoran. Anterior to the paracone, there is a strongly reduced and rounded cusp. Similar to the ?metacone, this cusp may be considered the ?preparacrista or the ?parastyle, following Solé *et al.* [61], and pending further studies on the evolution of the dentition in (early) Pinnipedimorpha.

P2–M1 are severely damaged, precluding detailed description. They are more elongate than P1, the lingual convexity is less pronounced, and the labial concavity is slightly more pronounced than in P1. P2–P4 are implanted slightly obliquely to the tooth row axis, with the distal extremity of P2 located labial to the mesial extremity of P3 and the distal extremity of P3 located labial to the mexial extremity of P4. P2–P4 are morphologically similar to P1, having a lowly-raised paracone and a ?metacone distal to it. The height of the cusp mesial to the paracone is strongly reduced but mesiodistally long and may be considered the ?preparacrista or the ?parastyle. Posterior to the ?metacone, there is a small protuberance that can be considered the ?postmetacrista or the ?metastyle.

The first upper molar (M1) is separated from and slightly smaller than the premolars. M1 is morphologically strongly similar to P1, and is clearly premolariform, having lost the trigonid morphology and the protocone that are still present in early stem pinnipedimorphs [46–49,52–54]. The prominence of the paracone and the ?metacone is greater than in the premolars.

The enamel of the postcanine teeth is ‘wrinkled’, as had been observed for *H. martini* by Amson & Muizon [8]. The protocone is not as prominent as in other Monachinae, but broad: contributing to the robust appearance of the teeth. The metacone is large, about half the size of the protocone. The paracone anterior to the protocone is strongly reduced. The robust upper dentition of *N. gaudini* is most similar to that of the extant *Monachus* and a number of extinct Monachinae, such as *H. martini* and *Pl. etrusca*, while extant Monachinae (except *Monachus*) have highly specialized teeth. The mandibles have been described for both *Afrophoca libyca* and *N. gaudini*, and both are geographically and possibly geochronologically close [7,36]. However, the state of preservation of the holotype, and only specimen, of *A. libyca* (ELNRP 2Z131) is poor, and the illustrated mandible of *N. gaudini* ([7]: fig. 6) is missing (Giovanni Bianucci 2017, personal communication). Therefore, formal comparison between both taxa is for now precluded.

***Monotherium*? *wymani* (Leidy, 1853)**

(Figure 3)

**Figure 3.**
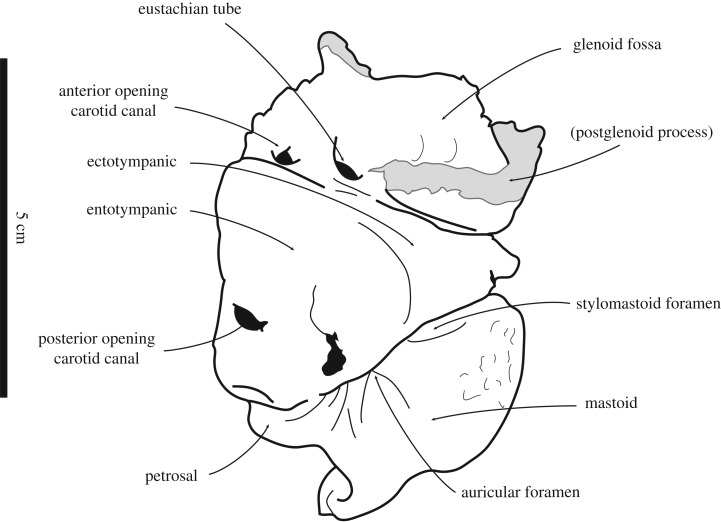
Line drawings of the holotype tympanic bulla MCZ 8741 of *Monotherium*? *wymani* (?Calvert Formation at Richmond, Virginia) in ventral view. After figures from Ray [15]. Broken and obliterated parts are indicated in grey. Scale bar equals 5 cm.

*Holotype*. MCZ 8741, left and right temporal bones, originally assigned to *Phoca wymani* by Leidy [62], ‘Tertiary’, Richmond, Virginia, USA.

*Type locality*. ‘Shockoe creek ravine near the base of Church Hill’ [63, p. 229], which is located in Richmond, Virginia, USA. Ray [15] provided evidence supporting Wyman's statement.

*Type horizon*. Ray [15] elaborated on the probable type horizon of MCZ 8741, concluding on the Calvert Formation. However, the Calvert Formation spans across the entire early Miocene and into the late middle Miocene (*ca* 23.03–13.8 Ma). At the Calvert Cliffs in Maryland, the oldest published record phocid fossils come from zone 10 of the Calvert Formation [64] which is dated to the early middle Miocene ([65]; and references therein). This renders a pre-middle Miocene age for MCZ 8741 less likely.

*Comments*. Originally presented as *Phoca wymani*, Ray [15] tentatively placed the original material of the species in *Monotherium*? *wymani*, as well as newly described specimens. The fossil record of *M.*? *wymani* is difficult to assess: The holotype MCZ 8741, a cranial fragment including the ear region and malleus, is valuable for the differentiation between different taxa of Phocidae [8,59], but the fossil record of Phocidae from the North Atlantic and Paratethys contains only a few ear regions [2]. Hence, the diagnostic value of fossil phocid ear regions is undermined. Additionally, a study of tympanic bullae of elephant seals, *Mirounga*, has shown that intraspecific variation is prominent [52]. Because other specimens attributed to *M.*? *wymani* only include disassociated and/or postcranial bones, Ray [15] could only tentatively assign them to *M.*? *wymani*. Moreover, the fossil record of referred specimens of *M.*? *wymani* does not include either humeri or femora, despite these bones being the most valuable postcranial bones for the identification of Phocidae [2,15]. The lack of humeri in the fossil record of *M.*? *wymani* precludes any comparison of the taxon with the original *Monotherium* humeri from the Miocene of Belgium (lectotype humerus of *Frisiphoca aberratum* IRSNB 1191-M266, and lectotype humerus of *Frisophoca affine* IRSNB 1118-M260 in this study). The holotype ear region of *M.*? *wymani* is moderately well inflated, yielding a tympanic bulla that is roughly triangular in ventral view, and the posterior carotid foramen is clearly visible in ventral view (figure 3). These characteristics clearly support the identification of MCZ 8741 as a monachine (e.g. [15,43]). However, because the genus *Monotherium* is restricted to its type species, *M. delognii*, and because this type species is restricted to its lectotype pelvis IRSNB 1153-M257a, b, the holotype specimen of *M.*? *wymani* is reidentified as a monachine of uncertain affinities. The other specimens tentatively referred to *M.*? *wymani* are USNM 187410 (partial right mandible, left ulna, and right tibia and fibula) and USNM 214625 (partial fibula). The trochlear notch of the ulna of USNM 187410 is very similar to that of the ulna IRSNB 1121-M261a, and both are considered Phocidae cf. *Frisiphoca affine*. Muizon [42] already noted the value of the shape of the trochlear notch of the ulna as a means to distinguish Phocidae to the generic level, also indicating similarities between the ulnae of Phocidae cf. *Frisiphoca* [*Monotherium*] *affine* (IRSNB 1121-M261a) and *Monotherium*? *wymani* with the ulnae of *Homiphoca capensis* and *Piscophoca pacifica* of South Africa and Peru, respectively. Ulna USNM 187410 can be considered as Phocidae cf. *Frisiphoca affine*. Contrastingly, we deem it inappropriate to identify the mandible and the partial tibia and fibula of USNM 187410 beyond the subfamily level. Ray [15] stated that the specimens came from one single block and are probably of one individual, but he implicitly expressed his doubt. Indeed, their association can be considered questionable in the absence of other preserved parts of the skeleton.

Pending the discovery of more complete fossil specimens of *Monotherium*? *wymani*, we propose to restrict the species *M*.? *wymani* to the holotype tympanic bulla (MCZ 8741), discarding the remainder of the fossil record proposed by Ray [15], because it cannot be compared to the holotype. The holotype of ‘*Phoca*’ *wymani* cannot be compared to the original *Monotherium* material from Belgium and Italy, i.e. the holotype skull of *Noriphoca gaudini*, the lectotype humeri of *Frisiphoca aberratum* and *Frisiphoca affine*, and the lectotype pelvis of *Monotherium delognii*. Apart from *M.*? *wymani*, tympanic bullae have been presented for three other Neogene seals from the North Atlantic: *Leptophoca proxima* [2], *Terranectes magnus* and *Terranectes parvus* [66]. However, Dewaele *et al*. [10] questioned the former designation of the skull of *L. proxima* to the species. And similarly, new research by Dewaele *et al*. (in preparation) questions whether the tympanic bullae referred to *T. magnus* and *T. parvus* can be securely attributed to these taxa. Thus, the holotype of *M.*? *wymani* cannot be compared to other contemporaneous Phocidae from the North Atlantic. Therefore, we consider *M.*? *wymani* to be a monachine of unknown affinities, pending the discovery of more complete specimens that can be attributed to the taxon. Despite being uncomparable to *Monotherium delognii*, we provisionally retain the genus name *Monotherium* with a question mark. It is unknown whether *M.*? *wymani* indeed belongs to the genus *Monotherium*. It is likely that the holotype will eventually be designated into another genus, but this cannot be ascertained based on the current fossil record. Contrastingly, *Monotherium gaudini* is given a new genus name in this study, based on the argumentation that the systematic comparison and phylogenetic analysis (see above and below) of the more complete type material places it as a stem monachine, while the phylogenetic affinities of the holotype of *M.*? *gaudini* with other Monachinae cannot be ascertained (see below). The current genus name *Monotherium* is preferred as placeholder over the genus name *Phoca,* which was used prior to Ray's redescription [15]. The name *Phoca* is currently restricted to *Phoca largha* and *Phoca vitulina*: two phocine seals that show no affinities with *M.*? *wymani* (see below).

**Subfamily Phocinae Gray, 1821**

**Genus *Frisiphoca* nov. gen.**

*LSID*. urn:lsid:zoobank.org:act:C4514A72-F792-4414-8130-2D595E413954

*Type species*. *Frisiphoca aberratum* (Van Beneden, 1876).

*Other included species*. *Frisiphoca affine* (Van Beneden, 1876).

*Diagnosis*. Identification as a phocid seal supported by the large development of the deltopectoral crest on the humerus. Identified as a phocine based on the presence of an entepicondylar foramen (also in *Homiphoca capensis*) and the overall slenderness. Differs from most Phocinae by having a very strongly reduced humeral neck (also in *Histriophoca fasciata*, *Leptophoca proxima* and *Pagophilus groenlandicus*). Differs from all Phocidae in the following unique combination of characteristics: lesser tubercle slightly below the level of the humeral head (also in *Devinophoca emryi*, *Le. proxima*, *Monachopsis pontica*, *Nanophoca vitulinoides*, *Pachyphoca chapskii*, *Pachyphoca ukrainica*, *Praepusa vindobonensis*, *Properiptychus argentinus* and *Sarmatonectes sintsovi*), transverse bar in bicipital groove (also in *Lobodon carcinophaga*, *Monachus monachus*, *Ommatophoca rossii*, *Piscophoca pacifica* and *Pliophoca etrusca*), deep fossa for *m. triceps brachii* distal to the humeral head (also in *Pi. pacifica*), and deltopectoral crest tapering smoothly distally (also in *Australophoca changorum*, *Acrophoca longirostris*, *Cryptophoca maeotica*, *De. emryi*, *Kawas benegasorum*, *Messiphoca mauretanica*, *Mo. pontica*, *Pachyphoca chapskii*, *Pachyphoca ukrainica*, *Pi. pacifica*, *Pl. etrusca*, *Pra. vindobonensis*, *Prophoca rousseaui*, *Properiptychus argentinus* and *S. sintsovi*).

*Etymology*. From the Latin pronoun ‘*Frisicum*’ and the Greek noun ‘*phoke*’. ‘*Frisicum*’ refers to the historical region of Frisia and the much smaller modern Dutch province with the same name. Here, the term is used in reference to the *Mare Frisicum*, the Latin name for the North Sea, alluding to the geographical origin of the two species of the genus listed here. ‘*Phoké*’ means ‘seal’.

*Comments*. Although *Frisiphoca* shares characteristics with both Monachinae and Phocinae (see diagnosis), the presence of an entepicondylar foramen is regarded as a characteristic uniting *Frisiphoca* with Phocinae. Unfortunately, this is not reflected in the phylogenetic analysis due to the poor scoring of the fragmentary fossil record of the genus.

**Figure 4.**
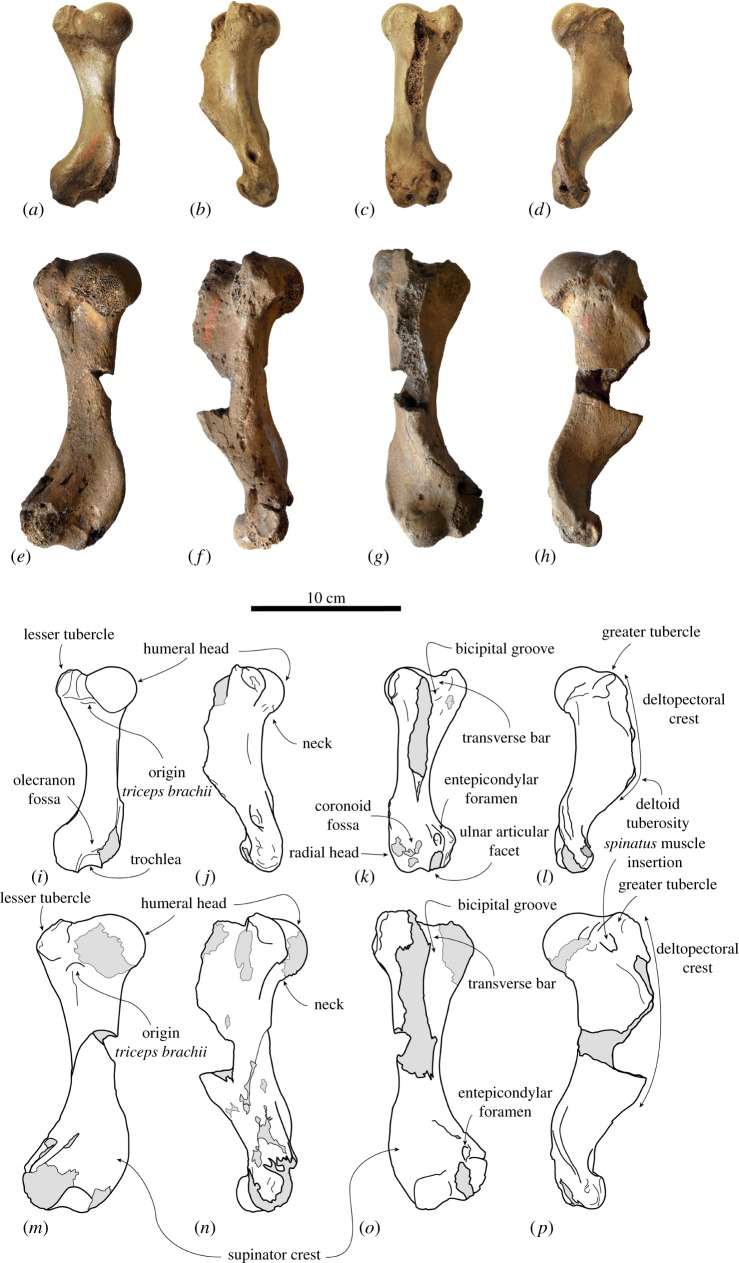
Lectotype right humerus IRSNB 1191-M266 of the stem phocine *Frisiphoca aberratum* from the ‘Diestian’ of the ‘third section’ at Borgerhout, Antwerp, in posterior (*a*), medial (*b*), anterior (*c*) and lateral (*d*) view. Lectotype right humerus IRSNB 1118-M260 of the stem phocine *Frisiphoca affine* from the ‘Diestian’ of the ‘third section’ at Deurne, Antwerp, in posterior (*e*), medial (*f*), anterior (*g*) and lateral (*h*) view. Corresponding labelled drawings of right humerus IRSNB 1191-M266 of *Frisiphoca aberratum* in posterior (*i*), medial (*j*), anterior (*k*) and lateral (*l*) view; and lectotype right humerus IRSNB 1118-M260 of *Frisiphoca affine* in posterior (*m*), medial (*n*), anterior (*o*) and lateral (*p*) view. Broken and obliterated parts are indicated in grey. Scale bar equals 10 cm.

***Frisiphoca aberratum* (Van Beneden, 1876)**

(Figure 4*a–d*, *i*–*l*)

*LSID*. urn:lsid:zoobank.org:act:E8382F26-74B1-4D16-AA5E-21F85821FF4D

*Lectotype*. IRSNB 1191-M266, partial right humerus. Van Beneden nor any subsequent author assigned a type specimen to *Frisiphoca* [*Monotherium*] *aberratum.* In the absence of more completely preserved material, we consider the humerus figured by Van Beneden ([17]; pl. 17, figs 1–4) the most diagnostic specimen to identify *F. aberratum*.

*Type locality*. Third section at Borgerhout (see original label with specimen), Antwerp, Belgium. The ‘third section’ follows Van Beneden's discretization of the nineteenth-century fortification constructions around the city of Antwerp, with the third section at Borgerhout being located northeast to the Borgerhout district of Antwerp ([9]: fig. 1; [10]: fig. 2) [9,10,17,67]. However, it should be noted that this type locality is derived from the original labels associated to the specimen. In his original publications Van Beneden [16,17] did not discuss the geographical provenance of individual specimens of the original fossil record of *Frisiphoca aberratum*.

*Type horizon*. Van Beneden (unpublished handwritten notes in the IRSNB archives) assigned the specimen IRSNB 1191-M266 to the ‘Diestien’ (Diestian). However, as mentioned above, the Diestian is currently considered an obsolete term and should not be used any more [20]. Different authors assign different ages and stratigraphic intervals to the Diestian (see [19]: table 1), but in general it is considered that the Diestian is roughly equivalent to the Deurne Sands Member of the Diest Formation. Louwye *et al*. [21] assigned a Messinian to Tortonian (late Miocene) age to the Diest Formation north of Antwerp and in the Campine area. In a more detailed description of the Neogene stratigraphy of the Antwerp area, Mourlon [68] mentions the occurrence of *Frisiphoca aberratum* (and possibly also *Frisiphoca* [*Monotherium*] *affine* and *Monotherium delognii*) in one of the strata he described. This unnamed stratum, composed of greenish glauconiferous sands overlays—also unnamed—darker green and black sands [68]. Extrapolating this to the current knowledge on the stratigraphy of the Antwerp area, these greenish glauconiferous sands most likely represent the Deurne Sands Member of the Diest Formation, while the underlying darker green sands and black sands represent the Antwerpen Sands Member of the Berchem Formation [69]. Therefore, it can be assumed that *F. aberratum* comes from the Deurne Sands Member of the Diest Formation (table 1).

*Diagnosis*. Medium-sized phocine, comparable in size to the harbour seal, *Phoca vitulina*. Differs from all Phocidae, including *Frisiphoca affine*, by the strong posteroproximal orientation of the humeral head, and differs from Phocinae, including *F. affine*, by the weak development of the supinator crest. Differs further from *F. affine* by the little medial curvature of the distal portion of the humerus, the shallow olecranon fossa (deeper in *F. affine*), and the smaller size (80.5% of length of humerus in *F. affine*; see table 2).

*Comments*. Although Van Beneden [17] assigned partially articulated specimens to *Frisiphoca* [*Monotherium*] *aberratum*, these specimens, including two partial pes associated with a baculum and three caudal vertebrae (IRNSB 1187-M273a-o); a phalanx and a fifth metatarsal (IRSNB 1188-M270a, b); a partial hind limb including a second and third metatarsal, a partial fibula, and an ectocuneiform (IRSNB 1189-M271a, b); and a thoriac and cervical vertebra (IRSNB 1132-M269a, b), bear little diagnostic value. Given the overall rarity of such bones in the fossil record, they cannot be compared with other extinct phocid taxa from the southern North Sea basin, and the lectotype humerus has been selected as the type specimen of *F. aberratum*, degrading the other specimens to Monachinae indet., Phocidae indet., or Phocinae indet. (see Supplementary Information 3). A sediment sample associated with the vertebrae IRSNB 1132-M269 was analysed biostratigraphically with dinoflagellate cysts for our study. The identification of this specimen as *F. aberratum* is questioned (this study; considered Phocidae indet.), but it should be mentioned that this sediment sample returned an age range from 7.25 Ma (latest Tortonian) to 3.7 Ma (late Zanclean), thus providing a minimum age interval for IRSNB 1132-M269 that does not contradict the stratigraphic assignment of the lectotype of *F. aberratum*.

Description and comparison

*Humerus* (figure 4*a*–*d*, *i*–*l*). In the absence of more complete, e.g. cranial, material, the humerus is the most diagnostic bone in Phocidae [2]. The humerus IRSNB 1191-M266 is overall well preserved, only missing part of the deltopectoral crest and portions of the distal epiphysis. The bone is straight and moderately slender. Ray [70] already noted that the humerus is relatively straight in some early Phocidae, such as *Leptophoca proxima*, as well as early stem pinnipedimorphs and terrestrial carnivorans, while most extinct and recent Phocidae have a more strongly curved humeral diaphysis. A measure for the ‘slenderness’ or ‘robustness’ of the humerus of Monachinae has been provided by Muizon & Bond [71]. This has been expanded to include Phocinae (table 3) and shows that the humerus in Phocinae is generally more slender than in Monachinae, although there is noticeable overlap. The humerus of *F. aberratum* is moderately slender and falls within the range observed in extinct and extant Phocinae (table 3).

The humeral head is small and strongly hemispherical. As in *Acrophoca longirostris*, *Hydrurga leptonyx* and Otariidae, the head faces relatively proximally (contra [42]), relatively more posteroproximal than in other Phocidae, including *F. aberratum*. Unlike other Phocinae, except the extinct *Leptophoca proxima* [10], and the extant *Histriophoca fasciata* and *Pagophilus groenlandicus*, the neck is poorly developed in the species of *Frisiphoca*.

The deltopectoral crest is slender in anterior view. Although incompletely preserved, it appears that the deltoid tuberosity must have been located approximately halfway the length of the bone. The bicipital groove bears a small but noticeable transverse bar, which is also observed in the Monachinae *Lobodon carcinophoca, Monachus* spp., *Ommatophoca rossii*, *Piscophoca pacifica* and *Pliophoca etrusca*, and in the phocine *Frisiphoca affine* [42]. The lesser and greater tubercles reach slightly below the level as the head (contra [42]; for the greater tubercle). Among Phocidae, most extant taxa (except *Monachus*) have a strongly developed lesser tubercle and a small greater tubercle. While this condition is variable among extant taxa, many early phocid taxa bear a relatively little-developed lesser tubercle (e.g. *Leptophoca proxima*, *Nanophoca vitulinoides*). Geologically younger extinct Phocidae (e.g. *Homiphoca capensis*, *Pliophoca etrusca*) tend to have a lesser tubercle that shows a degree of development intermediate between early extinct Phocidae and extant Phocidae [9]. On the lateral surface of the greater tubercle, the insertion area for the *infraspinatus* and *supraspinatus* muscles is well outlined. The deltopectoral crest is overall slender and tapers smoothly towards the coronoid fossa, distally, as is typical for many extinct monachines and phocines (see genus diagnosis) [10,42]. Just distal to the humeral head and lesser tubercle, on the posterior surface of the diaphysis, there is a prominent fossa for the origin of the *triceps brachii* muscles.

An entepicondylar foramen is present, which is a characteristic shared with other Phocinae; among Monachinae, only *Homiphoca capensis* has an entepicondylar foramen [42,58]. The supinator crest is reduced, as in Monachinae, but contrasting to other Phocinae, including *F. affine*. The medial epicondyle is broad and flaring, as in *F. affine*. The lateral margin of this supinator crest is rugose, providing an origin area for powerful manual extensor muscles. The medial epicondyle is broad at the distal extremity of the supinator crest and medial to the medial condyle. The olecranon fossa and coronoid fossa are strongly reduced, these regions being almost completely flat. When compared to other Phocidae (except *Homiphoca*), at the distal epiphysis the ulnar articular facet of the trochlea is prominent in relation to the radial capitulum. This contrasts with *F. affine*, in which the coronoid fossa is less reduced. Overall, the humerus of *F. aberratum* combines characters which are otherwise considered typically monachine, or typically phocine. The poorly developed supinator crest is considered a plesiomorphic character retained in Monachinae, while the presence of an entepicondylar foramen is regarded as a plesiomorphic character retained in Phocinae [8,58].

**Phocinae cf. *Frisiphoca aberratum***

(Figure 5*a*)

**Figure 5.**
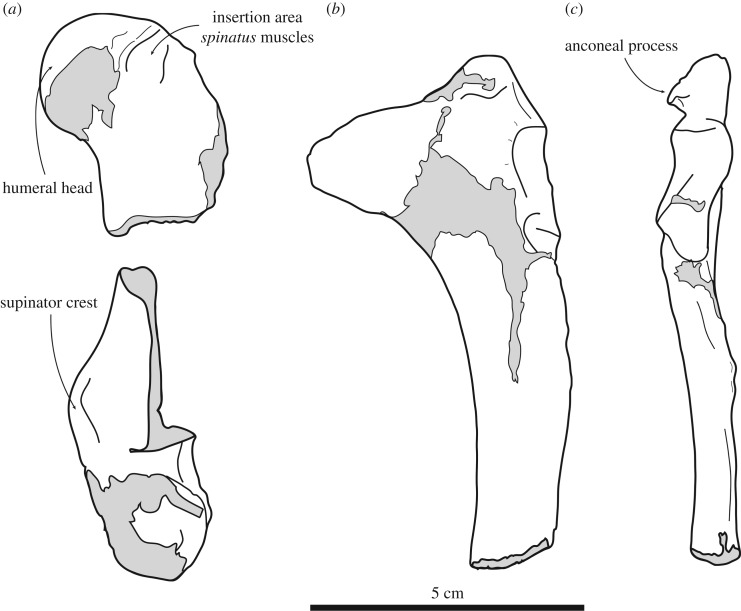
Line drawings of right humerus USNM 214625 (*a*) (Phocinae cf. *Frisiphoca aberratum*) (St Marys Formation of the Gay Head Greensands at Martha's Vineyard, Massachusetts) in lateral (*a*) view; and left ulna USNM 187410 (*b*,*c*) (Phocinae cf. *Frisiphoca affine*) (?Calvert Formation at Richmond, Virginia) in medial (*b*) and anterior (*c*) view. After figures from Ray [15]. USNM 214625 was considered ?*Monatherium aberratum*, and USNM 187410 was considered *Monotherium*? *wymani*. Broken and obliterated parts are indicated in grey. Scale bar equals 5 cm.

*Referred specimen*. USNM 214625, right humerus, Gay Head Greensand, Martha's Vineyard, Massachusetts, USA.

*Comments*. In his redescription of *Monotherium*? *wymani*, Ray [15] identified specimen USNM 214625, a partial humerus, as ?*Monotherium aberratum* ([15]: figs 8–11, subset 1). Despite the poor state of preservation, its general shape and size, and more specifically the proximal projection of the humeral head, the morphology of the greater tubercle, the shape of the insertion pit for the *spinatus* muscles, and the weak development of the supinator crest indicate similarities with the lectotype of *Frisiphoca aberratum* (figure 5*a*). However, given the poor state of preservation of the specimen, we deem it best to consider the specimen Phocidae cf. *Frisiphoca aberratum*. Dall [72] and Ray [15] considered the Gay Head Greensand to be part of the St Marys Formation, which is nowadays considered to be Tortonian (upper Miocene) in age (dinocyst zones DN 8 and 9 in Kidwell *et al*. [65]; 11.2–7.6 Ma from Köthe [73]).

***Frisiphoca affine* (Van Beneden, 1876)**

(Figure 4*e*–*h*, *m*–*p*)

*LSID*. urn:lsid:zoobank.org:act:75D28CBF-B02F-4727-ACD6-BFF2A7EB77EC

*Lectotype*. IRSNB 1118-M260, partial right humerus. Van Beneden nor any subsequent author assigned a type specimen to *Frisiphoca affine.* In the absence of cranial material, we consider the humerus the most diagnostic specimen to identify *F. affine*.

*Type locality*. ‘Third section at Deurne’ (see original label with specimen), Antwerp, Belgium. The third section at Deurne is located southwest along the Deurne district of Antwerp ([9]: fig. 2; [10]: fig. 1) [9,10,17,67]. This type locality is derived from the original labels associated to the specimen, while Van Beneden [17,18] did not discuss the geographical provenance of all specimens, restricting the description to ‘third section’.

*Type horizon*. Van Beneden (unpublished handwritten notes in the IRSNB archives) assigned IRSNB 1118-M260 to the ‘Diestien’ (Diestian). However, as mentioned above, the Diestian is currently considered an obsolete term and should not be used any more [19]; in general, it is considered roughly equivalent to the Deurne Sands Member of the Diest Formation, dated to Messinian to Tortonian (late Miocene; [21]). In addition to *Monotherium aberratum*, Mourlon [68] mentions the possible occurrence of *Monotherium affine* and *Monotherium delognii* in an unnamed stratum, composed of greenish glauconiferous sands overlays—also unnamed—darker green and black sands that most likely represents the Deurne Sands Member of the Diest Formation [69].

*Diagnosis*. Large phocine, comparable in size to the leopard seal, *Hydrurga leptonyx*, and larger than all other extant and extinct Phocinae, except *Erignathus barbatus*. The humerus of *Frisiphoca affine* differs from *Frisiphoca aberratum* by the strong medial curvature of the distal portion of the humerus, the moderately deep olecranon fossa (shallow in *F. aberratum*), and the larger size (humerus of *F. aberratum* is 80.5% the size of the humerus of *F. affine*, see table 2). The presence of a well-developed supinator crest on the humerus is another difference between *F. affine* (present) and *F. aberratum* (absent), commonly observed among Phocinae.

*Comments*. When Kellogg [18] elected *Monotherium delognii* as the type species for the genus *Monotherium*, he considered the differences between *M. delognii* and *Monotherium affine* minimal and regarded *M. affine* as a junior synonym to *M. delognii*. Based on the current fossil record, it is clear that, apart from the partial pelvis IRSNB 1153-M257a, b, none of the bones originally assigned to *M. delognii* bears enough diagnostic characteristics to identify it beyond the family level. This partial pelvis is selected as the lectotype of *M. delognii*, showing similarities to the pelvis of *Prophoca rousseaui*. However, it cannot be compared with *F. affine* due to the lack of a preserved pelvis in the known fossil record of *F. affine.* Therefore, we do not follow Kellogg [18] and do not regard *M. affine* as a junior synonym to *M. delognii*.

Description and comparison

*Humerus* (figure 4*e*–*h*, *m*–*p*). The lectotype humerus of *Frisiphoca affine* IRSNB 1118-M260 is the only known humerus for the species and it is moderately well preserved. The humerus of *F. affine* differs relatively little from the humerus of *Frisiphoca aberratum*. Overall, the humerus is also slender and straight, but it is noticeably longer than the humerus of *F. aberratum* (table 2). The incompleteness of the humerus precludes quantification of the ‘slenderness’ or ‘robustness’ as has been done for *F. aberratum* (table 3). However, the similar shape allows assuming similar robustness values for both *Frisiphoca* species. The humeral head of *F. affine* is strongly hemispherical. The bicipital groove is moderately wide and relatively open, i.e. the margins of the bicipital groove almost form a straight angle and are not U-shaped in section. The bicipital groove bears a little-developed but noticeable transverse bar. This transverse bar was also observed in a large number of Monachinae and *F. aberratum* [42]. The lesser tubercle does not reach the level of the head and the greater tubercle reaches the same level as the head. Among other Phocidae, comparable conditions have been observed in extinct taxa, such as the middle Miocene phocines *Leptophoca proxima* and *Pachyphoca* spp. In extant phocids only *Monachus* has a somewhat reduced lesser tubercle (see above). As in *F. aberratum*, the insertion area for the *infraspinatus* and *supraspinatus* muscles is well outlined on the lateral surface of the greater tubercle. Distally, the deltopectoral crest tapers smoothly towards the coronoid fossa. As already pointed out by Muizon [42], the posterior surface of the diaphysis bears a prominent fossa just distal to the humeral head and lesser tubercle, for the origin of the *triceps brachii* muscles. This was also observed in the South American stem lobodontins *Acrophoca* and *Piscophoca*, as well as in *F. aberratum* [42]. An entepicondylar foramen is present. The supinator crest is well developed, as in other Phocinae except *F. aberratum*.

**Phocidae cf. *Frisiphoca affine***

(Figure 5*b*,*c*)

*Referred specimens*. IRSNB 1121-M261a, right ulna, ‘Diestian’, third section at Borgerhout, Antwerp, Belgium. IRSNB 1126-M262, left astragalus, ‘Diestian’, third section at Borgerhout. IRSNB 1125-M263, right calcaneum, ‘Diestian’, third section at Borgerhout. USNM 187410, left ulna, Calvert Formation?, Richmond, Virginia, USA.

*Comments*. All Belgian specimens considered as Phocidae cf. *Frisiphoca affine* in this study had originally been identified and illustrated as *Monotherium affine* [17]. However, these specimens have been found isolated and are generally considered of very little diagnostic value. Another radius (IRSNB 1138-M267), astragalus (IRSNB 1144-M272) and calcaneum (IRSNB 1187-M273d) have originally been assigned to *Monotherium aberratum*, which are much smaller than the referred specimens IRSNB 1121-M261b, IRSNB 1126-M262, IRSNB 1125-M263. Therefore, the fossil record of phocids from the ‘Diestian’ includes the larger bones referred to in this section, as well as comparatively smaller radius, astragalus and calcaneum. The larger set can be assigned to Phocidae cf. *Frisiphoca affine*, based on the larger size of the specimens better matching *F. affine*, presumably from the same lithological unit. The American specimen of Phocidae cf. *Frisiphoca affine*, specimen USNM 187410, had previously been considered as *Monotherium*? *wymani* [15].

Description and comparison of the Belgian material

**Figure 6.**
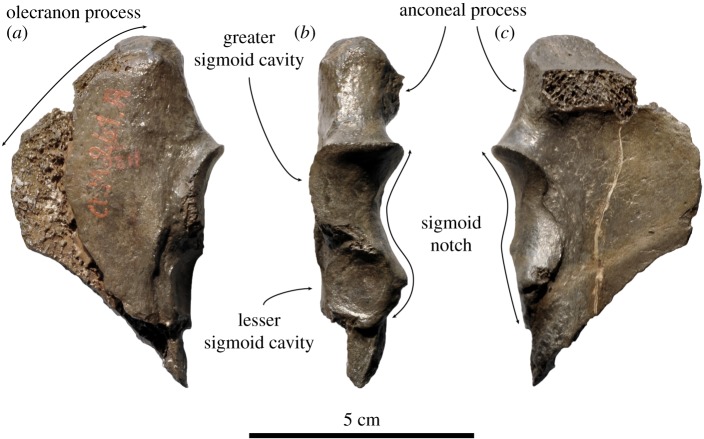
Right ulna IRSNB 1121-M261a assigned here to Phocidae cf. *Frisiphoca affine* (originally *Monatherium affine* by Van Beneden [17]) from the ‘Diestian’ of the ‘third section’ at Borgerhout, Antwerp, in lateral (*a*), anterior (*b*) and medial (*c*) view. Scale bar equals 5 cm.

*Ulna* (figures 5*b*,*c* and 6). The anteroproximal portion of a right ulna is preserved (IRSNB 1121-M261a) (figure 6). The anconeal process is located relatively more distal on the proximomedial surface of the olecranon process than it is in any other phocid, except specimen USNM 187410 (figure 5*b*,*c*), formerly assigned to *Monotherium*? *wymani* [15]. This yields a strongly sloping appearance for the anconeal process. The prominence of this anconeal process is similar to that in other Phocinae, while it is generally much reduced in Monachinae (except *Homiphoca*) [57]. The greater sigmoid cavity for articulation with the humerus is mushroom-shaped, with the upper, greater facet facing anteriorly. Gradually curving around the sigmoid notch, this facet transits distally in a smaller facet facing medially. The lesser sigmoid cavity (=radial notch) is located anterodistally of the greater sigmoid cavity. This cavity is circular, flat, and faces anterolaterally. Again, this matches well *M.*? *wymani*, but it should be highlighted that little attention has historically been given to the description of the shape of the sigmoid notch of fossil Phocidae. Published drawings and descriptions have shown appreciable differences in the shape of the sigmoid notch among extant Phocinae [59], but no detailed description has been provided for Monachinae [42,74]. Overall, the position of the anconeal process suggests that the ulna is phocine. However, being only one characteristic combined with the poor state of preservation of the specimens raises doubt on the validity of this assumption. Therefore, it is safer to consider the specimens Phocidae cf. *Frisiphoca affine*.

**Figure 7.**
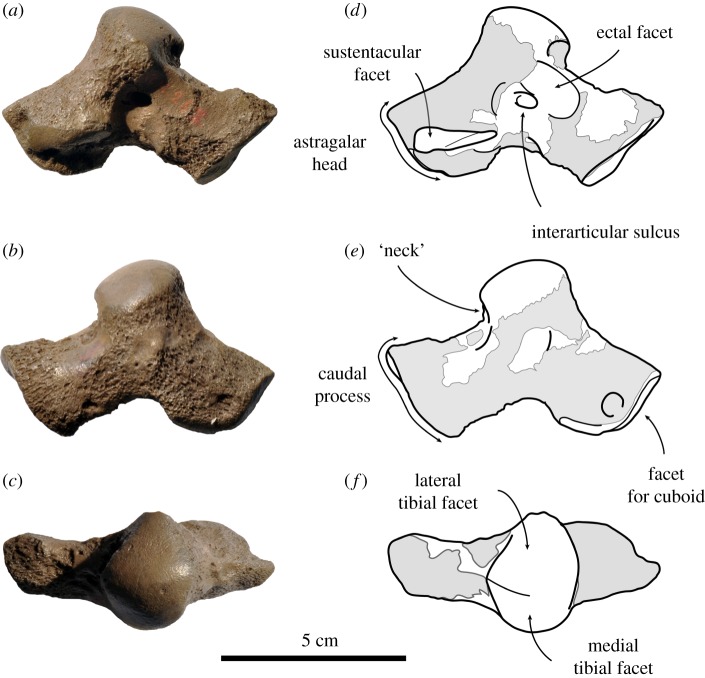
Left astragalus IRSNB 1126-M262 assigned here to Phocidae cf. *Frisiphoca affine* (originally *Monatherium affine* by Van Beneden [17]) from the ‘Diestian’ of the ‘third section’ at Borgerhout, Antwerp, in lateral (*a*), medial (*b*) and dorsal (*c*) view. Corresponding labelled drawings of IRSNB 1126-M262 in lateral (*d*), medial (*e*) and dorsal (*f*) view. Broken and obliterated parts are indicated in grey. Scale bar equals 5 cm.

*Astragalus* (figure 7). One isolated left astragalus (IRSNB 1126-M262) can very tentatively be assigned to Phocidae cf. *Frisiphoca affine*. Although it had been found isolated, at an absolute length of 75.5 mm (table 4), it is noticeably larger than the isolated specimen originally assigned to *Monotherium aberratum*, IRSNB 1144-M272 (considered Phocidae indet. in this study, see Supplementary Information 3), which has an estimated absolute length of 5.62 mm, and much larger than the adult male pes originally assigned to *M. aberratum* (IRSNB 1187-M273; also Phocidae indet. in this study, see Supplementary Information 3). In addition, a number of differences can be observed, separating this astragalus from the indeterminate phocid astragalus IRSNB 1144-M272.

The tibial facet is small, and proportionally smaller than in IRSNB 1144-M272 and other phocid astragali. The lateral and medial tibial facets form a right angle in proximal view, as in *Monachus* sp., Phocinae and *Piscophoca pacifica*; but this angle is slightly larger than in IRSNB 1144-M272. In lateral view, the tibial facet is convex. The tibial facet is separated from the caudal process by a ‘neck’, which is more pronounced than in other phocids, except *Acrophoca longirostris*. The caudal process is long, which is a typically phocid characteristic [58], and plantardorsally elongate, but mediolaterally not as thick as in astragalus IRSNB 1144-M272. The dorsoplantar height of the caudal process of IRSNB 1126-M262 is 30.0 mm, and the mediolateral width of the process is 16.6 mm. The dorsoplantar height of the caudal process of IRSNB 1144-M272 cannot be measured due to the poor state of preservation, but the mediolateral width is 18.5 mm, which is somewhat wider than in IRSNB 1126-M262. Although the proximodistally elongate ectal facet is not completely preserved, it appears proportionally thicker and less elongate than in IRSNB 1144-M272. However, the elongation of the ectal and sustentacular facets is marked, uniting both specimens with *Australophoca changorum*, *Monachus* sp., Phocinae and *Piscophoca pacifica*. The elongate sustentacular facet is well developed and highly raised over the astragalar head. This facet is convex and facing ventrolaterally; it is separated from the ectal facet by an interarticular sulcus. This sulcus forms a distinct oval fossa just ventroproximally of the ectal facet. Although this sulcus is present in other Phocidae as well [57], it only forms a deep and narrow fossa in IRSNB 1126-M262. The sustentacular facet is separated from the facet for the navicular. The facet for the navicular is plantardorsally elongate and runs along the entire distal margin and the distal portion of the plantar margin of the astragalus. The long axes of the caudal process and of the head form an obtuse angle of approximately 120°, making the astragalus arched in lateral view, as in extant Lobodontini. Given the strong similarities between the astragalus in Phocinae and some Monachinae, it is difficult to elucidate whether IRSNB 1126-M262 is monachine or phocine.

**Figure 8.**
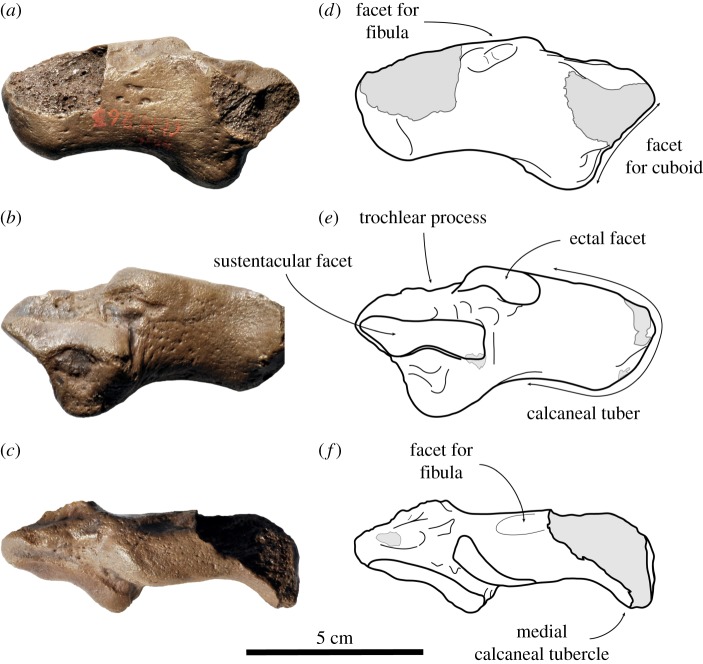
Right calcaneum IRSNB 1125-M263 assigned here to Phocidae cf. *Frisiphoca affine* (originally *Monatherium affine* by Van Beneden [17]) from the ‘Diestian’ of the ‘third section’ at Borgerhout, Antwerp, in lateral (*a*), medial (*b*) and dorsal (*c*) view. Corresponding labelled drawings of IRSNB 1125-M263 in lateral (*d*), medial (*e*) and dorsal (*f*) view. Broken and obliterated parts are indicated in grey. Scale bar equals 5 cm.

*Calcaneum* (figure 8). Similar to the astragalus, one isolated right calcaneum (IRSNB 1125-M263) can very tentatively be assigned to Phocidae cf. *Frisiphoca affine*, largely based on its relatively large size in comparison to another calcaneum from the ‘Diestian’ of Antwerp that was originally assigned to *Monotherium aberratum* (IRSNB 1187-M273d; Phocidae indet. this study, see Supplementary Information 3). Although it has been found isolated, the specimen is much larger than the adult male calcaneum IRSNB 1187-M273d (see Supplementary Information 3: figure S2*a*–*c*), precluding the possibility of sexual dimorphism as an argument to group both specimens in the same species. The total length of calcaneum IRSNB 1125-M263 is 78.8 mm, while the total length of calcaneum IRSNB 1187-M273d is only 51.2 mm (table 5; Supplementary Information 1: table S3). Furthermore, both calcanea differ morphologically. In lateral view, the calcaneum IRSNB 1125-M263 is distally much wider than it is proximally. However, the calcaneal tuber is plantardorsally relatively thicker in IRSNB 1144-M272 than in IRSNB 1125-M263. Muizon ([42]: table 7) employed the ratio of the plantardorsal height versus the total length of the calcaneum as a means to separate extant Lobodontini (high ratio; greater than 0.55) from other Monachinae (intermediate ratio) and Phocinae (low ratio; less than 0.50). For IRSNB 1125-M263, this ratio equals 0.503 (39.3 mm : 78.2 mm), at the boundary between Monachinae and Phocinae. The variability in dimensions is in correspondence to the differing length of the calcaneal tuber among Phocidae: the calcaneal tuber of IRSNB 1125-M263 is very long, as in Phocinae, Monachini and extinct Phocidae; but contrasts with extant Lobodontini, where the calcaneal tuber is short. Muizon [42] considered a long calcaneal tuber to be a plesiomorphic characteristic. IRSNB 1125-M263 bears a prominent medial process at its proximal end that is well developed, as in IRSNB 1187-M273d. Halfway on the dorsolateral margin of the calcaneum, there is an oval, concave facet for the articulation with the fibula. Such a facet has also been observed in IRSNB 1187-M273d, and is generally considered more prominent in Monachinae than in Phocinae [42]. The trochlear process extends across the dorsal surface of the calcaneum, anterior to the ectal facet (=proximal astragalar facet). The astragalar articular facets are relatively long, as in Phocinae. The sustentacular facet (=distal articular facet) is slightly less slender than in IRSNB 1187-M273d and the curvature of the facet in medial view is less pronounced. The ectal facet (=proximal articular facet) is oriented anterodorsally--posteroventrally, but nearly horizontal, and is shorter than in IRSNB 1187-M273d and slightly thicker. In IRSNB 1125-M263, the length of the ectal facet is 26.6% of the total length (20.8 mm : 78.2 mm), and in IRSNB 1187-M273d, the ectal facet is 28.3% of the total length (14.5 mm : 51.2 mm). The height-to-length ratio of the ectal facet is 41.3% in IRSNB 1125-M263 (8.6 mm : 20.8 mm) and 37.9% in IRSNB 1187-M273d (5.5 mm : 14.5 mm). Anteriorly, the sustentacular facet transits into the cuboid facet. The concave and lozenge-shaped cuboid facet is higher than wide and, contrasting to IRSNB 1187-M273d, not restricted to the dorsal two-thirds of the distal margin of the calcaneum. Although sharing a number of characteristics with extant Phocinae and not with extant Monachinae, because extinct Monachinae and Phocinae tend to exhibit an overall intermediate morphology [42], it is impossible to confidentially assign this specimen to either of both subfamilies and its comparison with *F. affine* is largely based on its size.

**Phocidae indet.**

***Monotherium* Van Beneden, 1876**

*Type species and only included species*. *Monotherium delognii* Van Beneden, 1876.

*Diagnosis*. As for the species

***Monotherium delognii* Van Beneden, 1876**

(figure 9)

**Figure 9.**
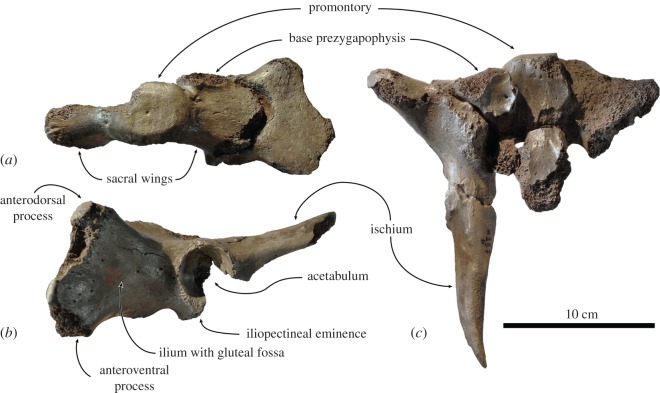
Partial pelvis IRSNB 1153-M257a, b including a sacrum (a) and a left innominate (b), assigned to *Monatherium delognii* (Van Beneden [17]) from the ‘Diestian’ of the ‘third section’ at Deurne, Antwerp in anterior (*a*), left lateral (*b*) and dorsal (*c*) view. Scale bar equals 10 cm.

*Lectotype*. IRSNB 1153-M257a, b, partial sacrum including the sacral wings and the bodies of the first and second sacral vertebrae, and the associated left innominate represented by the ilium and the acetabular branch of the ischium, originally assigned to *Monatherium delognii* by Van Beneden ([17]: pl. 16, figs 5, 6), ‘Diestian’, third section at Deurne, Antwerp, Belgium.

**Figure 10.**
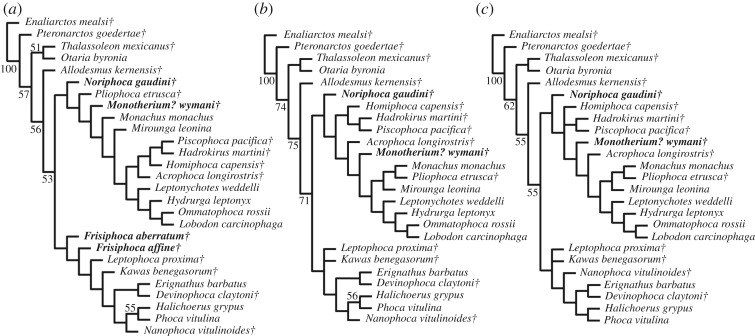
Phylogenetic tree resulting from the (*a*) first, (*b*) second and (*c*) third analysis. (*a*) The first analysis includes *Frisiphoca aberratum*, *Frisiphoca affine*, *Monotherium*? *wymani* and *Noriphoca gaudini*. 50% majority consensus tree of 29 most parsimonious trees without equal weighting of homoplastic characters. (*b*) The second analysis includes *M.*? *wymani* and *N. gaudini*. 50% majority consensus tree of 75 most parsimonious trees without equal weighting of homoplastic characters. (*c*) The third analysis includes *M.*? *wymani* and *N. gaudini* (*a*). 50% majority consensus tree of 174 most parsimonious trees with equal weighting of homoplastic characters. *Frisiphoca aberratum*, *F. affine*, *M.*? *wymani* and *N. gaudini* highlighted in bold. Extinct taxa are indicated by a dagger. All bootstrap values equal to or higher than 50 (after 10 000 replicates) are shown.

*Type locality*. Third section at Deurne, Antwerp, Belgium. The ‘third section’ follows Van Beneden's discretization of the nineteenth-century fortification constructions around the city of Antwerp, with the third section at Deurne being located southwest to the Deurne district of Antwerp ([9]: fig. 2; [10]: fig. 1) [9,11,17,67]. It should be noted that this type locality is derived from the original labels associated to the specimen. In his original publications Van Beneden [16,17] did not discuss the geographical provenance of individual specimens of the original fossil record of *Monotherium delognii*.

*Type horizon*. Van Beneden (unpublished handwritten notes in the IRSNB archives) assigned the specimen IRSNB 1153-M257a, b to the ‘Diestien’ (Diestian). However, as mentioned above, the Diestian is currently abandoned, with different authors providing different ages and stratigraphic intervals for the Diestian ([19]: table 1). Generally, it is considered that the Diestian is equivalent to the Deurne Sands Member of the Diest Formation. Louwye *et al*. [21] assigned a Tortonian (late Miocene; 8.8–11.4 Ma) age to the Diest Formation north of Antwerp and in the Campine area.

*Diagnosis*. Large phocid, comparable in size to the monachine *Leptonychotes weddelli*, larger than the extinct phocine *Prophoca rousseaui*. Differences from other Phocidae (except *Prophoca rousseaui*) in: straight horizontal ventral margin of the sacral wings, wide base for the prezygapophysis of S1, the anterior offset of the promontory to the sacral wings, an elongate ilium, weak lateral eversion of the ilium (also in Monachinae, *Erignathus barbatus* and *Kawas benegasorum*), and the weak development of a gluteal fossa on the ilium (also in Monachinae, *E. barbatus* and *K. benegasorum*). Differs from *P. rousseaui* by a dorsoventral compression of the promontory of the sacrum and more rounded lateral margins of the sacral wings.

*Comments*. Neither Van Beneden [16,17] nor subsequent researchers (e.g. [18]) assigned a type specimen to *Monotherium delognii*. Of all specimens assigned to *M. delognii*, the specimen IRSNB 1153-M257a, b is the least unsatisfactory in terms of diagnostic value. As mentioned above, Van Beneden [16,17] did not assign a type species to the genus *Monotherium*; later, Kellogg [18] considered *Monotherium delognii* as the type species, giving it page priority over *Monotherium affine* and *Monotherium aberratum*. Originally, Van Beneden [17] assigned a much more extensive number of specimens to *M. delognii*, also including vertebrae (IRSNB 1108, IRSNB 1108-M255a, b, IRSNB 1216, IRSNB 1217, IRSNB 1217-M256a), a radius (IRSNB 1139), a fibula (IRSNB 1149), phalanges (IRSNB 1217-M256b, IRSNB 1227) and indeterminate remains (IRSNB 1115). The state of preservation of these specimens, composed of moderately to poorly preserved disassociated bones, does not allow identification to a specific phocid taxon; they are, hence, considered indeterminate monachine, phocid or phocine specimens (Supplementary Information 3). From the current study it is evident that the lectotype of *M. delognii* shows similarities with the pelvis of the phocine *Prophoca rousseaui* [10]. However, the size of the pelvis matches well the size of the lectotype humerus IRSNB 1118-M260 of *Frisiphoca affine* (see below). Because no pelvis is currently known for the latter species, no further comparison can be done. Due to its similarities to *P. rousseaui*, this pelvis of *M. delognii* is tentatively considered a phocine pelvis, pending the discovery of more complete specimens and a detailed phylogenetic analysis.

Description and comparison

*Sacrum* (figure 9). The sacrum IRSNB 1153-M257a is only very partially preserved: only the sacral wings, and the bodies of the first and second sacral vertebrae are incompletely preserved and severely abraded, inhibiting a detailed description. The promontory is dorsoventrally slightly compressed (43.9 mm : 60.9 mm). The sacral wings (*alae*) are strongly laterally enlarged, but not as pronounced as in *Prophoca rousseaui* or some other phocids [10]: the transverse width across the wings is 2.70 times the lateral width across the promontory (164.4 mm : 60.9 mm) (3.31× in *P. rousseaui*, [10]). Although past studies indicated that this ratio is higher in Monachinae than in Phocinae [42], Dewaele *et al*. [10] showed that there is considerable overlap between the ratio ranges of Monachinae and Phocinae, preventing clear distinction between both subfamilies. The lateral margins of the sacral wings are badly preserved, but they appear to have been originally rounded. The ventral margins of the sacral wings are remarkably straight horizontally, as in *P. roussseaui*, but unlike other Phocidae. Other similarities with *P. rousseaui* are the dorsal and posterior offset of the wings in relation to the promontory, and the wide base of the prezygapophysis.

*Innominate* (figure 9). The partial left innominate IRSNB 1153-M257b associated with the partial sacrum IRSNB 1153-M273a is noticeably larger than the innominates IRSNB 1192-M276 and IRSNB M2234 (both assigned to *Prophoca rousseaui* [10]). Similar to what we observe for the sacrum, the innominate IRSNB 1153-M273 strongly resembles that of *P. rousseaui* (most noticeably IRSNB 1192-M276). The ilium is strongly elongate in comparison with other Phocidae, except the monachines *Monachus monachus* and *Piscophoca pacifica*, and the phocine *P. rousseaui*. Following the procedure of Dewaele *et al*. [9] to quantify the lateral eversion of the ilium of Phocidae, the angle of the ilium to the postacetabular region of innominate IRSNB 1153-M257b is 63.4°. This falls within the range observed for Monachinae (average 65.6°), but at the lower end of the range of extant Phocinae (average 74.6°) [9]. The lateral eversion of the ilium and the degree of development of the gluteal fossa are similar to that in Monachinae, the extinct phocines *Kawas benegasorum* and *P. rousseaui*, and the extant phocine *Erignathus barbatus*. As in *P. rousseaui* the shape of the iliac crest is slender, with the anteroventral process located anterior to the level of the anterodorsal process. The posterodorsal and posteroventral processes are well developed as well. The iliopectineal eminence is very incompletely preserved, but appears well developed. Lastly, a deep acetabulum is shared with *Monachus* spp. and Phocinae. Contrasting to *P. rousseaui*, the ilium is not as markedly triangular in outline in IRSNB 1153-M273b. It should be noted that Dewaele *et al*. [10] suggested some degree of sexual dimorphism in *P. rousseaui*, considering the larger IRSNB 1192-M276 as a hypothetical male and IRSNB M2234 as a female. Given the observed number of differences and the incompleteness of the fossil record of *M. delognii*, it is not replaced into *Prophoca*, pending the discovery of more complete specimens.

Phylogenetic analyses

*First analysis* (figure 10*a*). A first phylogenetic analysis (*k*-value of the Goloboff criterion set at three) includes all three redescribed and reassigned taxa *Frisiphoca aberratum*, *Frisiphoca affine* and *Noriphoca gaudini*, as well as *Monotherium*? *wymani*. The pinnipedimorphs *Enaliarctos mealsi* and *Pteronarctos goedertae*, the otariids *Otaria byronia* and *Thalassoleon mexicanus*, and the desmatophocid *Allodesmus kernensis* are selected as outgroups. This analysis resulted in 29 most parsimonious phylogenetic trees (50% majority consensus tree figure 10*a*) with score −60.10 (tree length 220) after 133 718 tried rearrangements. Consistency index (CI) is 0.45, homoplasy index (HI) is 0.55, retention index (RI) is 0.69, and rescaled consistency index (RC) is 0.31. Higher level phylogenetic relationships correspond better with previously published phylogenetic analysis, returning *Enaliarctos mealsi* and *Pteronarctos goedertae* as stem Pinnipedimorpha, *Allodesmus kernensis* (Desmatophoca), Otariidae and Phocidae as Pinnipedia, and *A. kernensis* and Phocidae as Phocoidea [58,75]. *Kawas benegasorum* and *Leptophoca proxima* are returned as stem Phocinae, as has been shown before [9,10]. However, *Devinophoca claytoni* and *Nanophoca vitulinoides* are not returned as stem Phocinae, contrasting with a recent analysis by Dewaele *et al*. [9]. Historically, there has been little consensus on the phylogenetic relationships of extant and extinct Monachinae [6,8,76]. This first phylogenetic analysis returns *F. aberratum* and *F. affine* as stem Phocinae (figure 10*a*), branching off prior to *K. benegasorum* and *L. proxima*. *Monotherium*? *wymani* and *Noriphoca gaudini* are returned as stem Monachinae (figure 10*a*). Both *Frisiphoca aberratum* and *Frisiphoca affine* are very incompletely scored (7/80 characters) and share typical features with both Monachinae and Phocinae (see description), which makes it difficult to clarify phylogenetic affinities. Indeed, a test to elucidate the bootstrap support for this analysis resulted in poorly supported phylogenetic relationships among all Phocidae included in the analysis.

*Second analysis* (figure 10*b*). A second phylogenetic analysis (with down-weighting homoplastic characters and *k*-value of the Goloboff criterion set at three) excludes both *Frisiphoca* species, but includes *Monotherium*? *wymani* and *Noriphoca gaudini*. This analysis resulted in 75 most parsimonious phylogenetic trees (50% majority consensus tree, figure 10*b*) with score −60.21 (tree length 217) after 331 705 tried rearrangements. CI is 0.46, HI is 0.55, RI is 0.69 and RC is 0.32. The second analysis returns extinct Monachinae from South America (*Acrophoca longirostris*, *Hadrokirus martini* and *Piscophoca pacifica*) and South Africa (*Homiphoca capensis*) as stem Monachinae, while the first analysis returned them as a clade nested within crown Monachine. As in the first analysis (figure 10*a*), *M.*? *wymani* and *N. gaudini* are returned as stem monachines, with *N. gaudini* being the earliest branching stem monachine included in the analysis.

*Third analysis* (figure 10*c*). The same analysis as the second analysis (see above), but without down-weighting homoplastic characters, resulted in 174 most parsimonious phylogenetic trees (50% majority consensus tree, figure 10*c*) with score 216 after 727 267 tried rearrangements. CI is 0.46, HI is 0.54, RI is 0.69 and RC is 0.32. The topology of this analysis is largely similar to the topology of the second analysis (figure 10*b*), except for the phylogenetic position of *Nanophoca vitulinoides* among Phocinae and the phylogenetic position of *Homiphoca capensis* among Monachinae. The phylogenetic relationships of *M.*? *wymani* and *N. gaudini* are identical to the relationships observed in the second analysis. *Noriphoca gaudini* is again returned as the earliest branching stem monachine.

**Figure 11.**
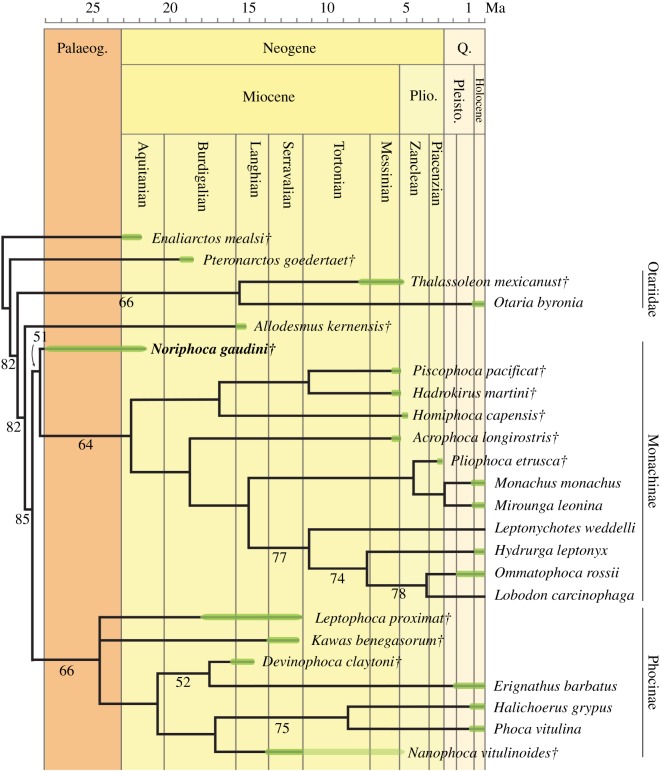
Phylogenetic tree resulting from the fourth analysis, excluding *Frisiphoca aberratum*, *Frisiphoca affine* and *Monotherium*? *wymani*. 50% majority consensus tree of the six most parsimonious trees. The age ranges for extinct OTUs are expressed as a green bar over each relevant terminal branch. Bootstrap values exceeding 50% are indicated on the relevant branches. Geochronological ages for the included species, whenever fossil or subfossil specimens have been documented: *Acrophoca longirostris* [42], *Allodesmus kernensis* [76] *Enaliarctos mealsi* [52], *Devinophoca claytoni* [30], *Erignathus barbatus* [77,78], *Hadrokirus martini* [8], *Halichoerus grypus* [79], *Homiphoca capensis* [80], *Hydrurga leptonyx* [18,81], *Kawas benegasorum* [82], *Leptophoca proxima* [10], *Mirounga leonina* [83], *Monachus monachus* [84], *Nanophoca vitulinoides* [9], *Noriphoca gaudini* (this study), *Ommatophoca rossii* [81], *Otaria byronia* [85], *Phoca vitulina* [79], *Piscophoca pacifica* [8], *Pliophoca etrusca* [6], *Pteronarctos goedertae* [54] and *Thalassoleon mexicanus* [55]. Extinct taxa are indicated by a dagger. *Noriphoca gaudini* indicated in bold.

*Fourth analysis* (figure 11). The fourth phylogenetic analysis excludes *Frisiphoca* and *Monotherium*? *wymani* and focuses on elucidating the phylogenetic relationships of *Noriphoca gaudini*. The analysis with down-weighting homoplastic characters and *k*-value of the Goloboff criterion set at three resulted in three most parsimonious phylogenetic trees (50% majority consensus tree, figure 11) with score −57.23 (tree length 246) after 15 494 tried rearrangements. CI is 0.40, HI is 0.60, RI is 0.62 and RC is 0.25. The analysis returns the same topology as for the second analysis (figure 10*b*), but with the exclusion of *M.*? *wymani*. *Noriphoca gaudini* is the first stem monachine to branch off the Monachinae clade. A bootstrap value of 51 supports the inclusion of *N. gaudini* among Monachinae. A complete list of the apomorphies that resulted from the phylogenetic analysis is provided as electronic supplementary material (Supplementary Information 1: figure S1 and table S5). In the phylogenetic analysis, the identification of *N. gaudini* is supported by one unequivocal autapomorphy: the ventral edge of the zygomatic arch is level with the alveolar plane (character 13, state ‘0’ to ‘1’).

*Fifth analysis*. Rerunning the fourth analysis without down-weighting homoplastic characters resulted in six most parsimonious phylogenetic trees with tree length 216 after 24 773 tried rearrangements. CI is 0.46, HI is 0.54, RI is 0.69 and RC is 0.32. The topology of this analysis differs from the fourth analysis for the Phocinae, with the clade composed of *Kawas benegasorum *+* Leptophoca proxima* returned as a sister clade to all other included Phocinae, and with *Nanophoca vitulinoides* returned as the last stem phocine before crown Phocinae. However, the phylogenetic position of *Noriphoca gaudini* as the earliest-branching stem monachine is unchanged.

## 6. Discussion

### 6.1. Systematic palaeontology

Prior to our reassessment of the extinct phocid *Monotherium*, this genus included five species: *Monotherium aberratum*, *Monotherium affine*, *Monotherium delognii*, *Monotherium gaudini* and *Monotherium*? *wymani*. Our study strongly impacts this content, questioning the validity of some species and reassigning others to different genera in different subfamilies. Following the current study, *Monotherium* is split in three different genera, including four species. The monachine *M.*? *wymani* cannot be compared to *Monotherium delognii*, the type species of *Monotherium* with unknown subfamilial attributions. However, the holotype of *M.*? *wymani* is two associated tympanic bullae, which are considered diagnostic in Phocidae. Hence, the redescription of *M.*? *wymani* is left *in limbo*, pending the discovery of more complete specimens that will allow comparison with other extinct Phocidae. *Monotherium gaudini* is identified as the earliest branching monachine seal. This is supported by multiple phylogenetic analyses. *Monotherium gaudini* is renamed to *Noriphoca gaudini*. The fossil records of *M. aberratum* and *M. affine* are limited to their respective holotype humeri. Despite the incompleteness of their fossil records, a detailed redescription and one preliminary phylogenetic analysis return both as stem Phocinae. Both species are regrouped into the new genus *Frisiphoca*: *Frisiphoca aberratum* and *Frisiphoca affine*.

### 6.2. Stratigraphy

Prior to the current study, the stratigraphic position and geological age of *Noriphoca gaudini* was poorly known and no formal reinvestigation of the original stratigraphic data have been published since the original publication by Guiscardi in 1870 [7]. To date, it remains impossible to retrace the exact geographical and stratigraphic position of the specimen. However, the Eocene Santo Spirito and late Oligocene to Miocene Bolognano formations are the only geological formations outcropping at the approximate type locality of *N. gaudini*, 3 km east of Roccamorice, Italy. Guiscardi [7] stated that the specimen came from a bituminous layer, which corresponds to the *Lepidocyclina* Limestone of the Bolognano Formation, dated to the Oligocene or earliest Miocene (Aquitanian) ([33,35]; and references therein). Consequently, *N. gaudini* can be considered the oldest known phocid seal, older than the Burdigalian *Afrophoca libyca* from Libya ([36]; contra [30,31]).

*N. gaudini* is clearly a phocid (e.g. the tooth row is diverging posteriorly (character 31, state ‘0’ to ‘1’) and M2 is absent (character 42, state ‘0’ to ‘1’)) (Supplementary Information 1, table S5), *N. gaudini* is arguably one of the oldest known crown pinnipeds, being equally old as or older than *Desmatophoca brachycephala* from the Aquitanian of the northeast Pacific [46]. Formerly, Koretsky & Sanders [30] and Diedrich [31] presented allegedly older phocid seal specimens from the late Oligocene of South Carolina and the Lutetian (middle Eocene) of Germany, respectively. Although these specimens are clearly phocid as well, Dewaele *et al*. [10] formally questioned the validity of stratigraphy of both findings. *Noriphoca gaudini* is also of the same age as *Enaliarctos* spp. from the Oligocene and early Miocene of the northeast Pacific [53]. This ascertains a late Oligocene diversification of Pinnipedimorpha and that different families of Pinnipedia already existed during the late Oligocene--early Miocene.

Formerly, a Diestian age had been assigned to specimens of *Monotherium* from Belgium. However, the Diestian age has been abandoned more recently. A detailed reinvestigation of the literature allows assuming that *Frisiphoca aberratum*, *Frisiphoca affine* and *Monotherium delognii* all come from the Deurne Sands Member of the Diest Formation [17,68]. Louwye *et al*. [21], Louwye [22] and Louwye & De Schepper [23] concluded that the deposits of the Diest Formation (Deurne Sands Member) near the city of Antwerp are of late Tortonian age. This age is moderately well supported by dinoflagellate cyst biostratigraphy of a sediment sample (1132LDW-1102Lab) associated with specimen IRSNB 1132-M269 (formerly *M. aberratum*, currently Phocidae indet.), which yielded an age range of 7.25–3.7 Ma (latest Tortonian to late Zanclean).

Overall, the stratigraphy of North American specimens that have been related to *Monotherium* [15] remains poorly resolved. One partial humerus sharing affinities with *Frisiphoca aberratum* comes from the Gay Head Greensand, which is considered to be part of the St Marys Formation (Tortonian, upper Miocene) (Dinocyst zones DN 8 and 9 in Kidwell *et al*. [65]; dates from Köthe [73]; see also Dall [72] and Ray [15]). This roughly matches the late Tortonian age proposed for the Deurne Sands Member of the Diest Formation, from where *F. aberratum* comes. Contrastingly, the stratigraphic origin of an ulna showing affinities with *Frisiphoca affine* and of the holotype ear region of *Monotherium*? *wymani* is poorly resolved.

### 6.3. Phylogenetic analysis and palaeobiogeographical considerations

The phylogenetic relationships among the Phocinae in the cladistic analyses correspond with the previously published trees by Dewaele *et al*. [9,10], with *Leptophoca proxima* and *Kawas benegasorum* as stem Phocinae. The phylogenetic relationships of extant and extinct Monachinae are less well established and different previously published analyses show different topologies [6,8,76]: the specific relationships among the four lobodontin species remain poorly resolved [34,43,47,86], and there is no agreement whether extinct Monachinae from the Southern Hemisphere form a distinct clade [8] or not [6,87], or whether they are stem Monachinae or not.

The phylogenetic trees obtained here clearly show that the extremely fragmentary fossil record of *Frisiphoca aberratum* and *Frisiphoca affine* precludes any detailed phylogenetic analysis and that it is for now impossible to resolve the subfamilial affinities of these taxa based on the current phylogenetic analysis. However, the presence of an entepicondylar in the lectotype humeri of *F. aberratum* and *F. affine* supports assigning the genus *Frisiphoca* to the Phocinae subfamily. *Monotherium*? *wymani*, on the other hand, is returned as a stem monachine, but the holotype is very incompletely coded (7/80 characters), and the exact phylogenetic relationships vary between different analyses (figure 10*b*,*c*) and remain unclear. Therefore, *M.*? *wymani* is considered a monachine of unknown phylogenetic affinities.

*Noriphoca gaudini* is a stem monachine, a result that agrees well with its geological age. Assuming that the type specimen of *N. gaudini* had indeed been collected from the bituminous layers of the *Lepidocyclina* Limestone of the Bolognano Formation, this species can be dated to the Oligocene or earliest Miocene (Aquitanian) ([33,35]; and references therein), which is older than the Burdigalian (*ca* 19 Ma) *Afrophoca libyca* from Libya [36]. Consequently, our phylogenetic analysis, together with the presence of *A. libyca* in the early Miocene Mediterranean, may suggest a Mediterranean origin for the subfamily Monachinae or even the entire family Phocidae. Ancestral non-phocid pinnipedimorphs were all restricted to the North Pacific realm [4,44–55]. The oldest pinnipedimorphs are *Enaliarctos* spp. from the late Oligocene, and the previously oldest known crown pinniped is *Desmatophoca brachycephala* from the Aquitanian of the northeast Pacific. It is broadly accepted that the direct ancestors of Phocidae crossed the Central American Seaway, with an origin of Phocidae along the east coast of North America ([4]; contra [88]). However, no fossils of these stem Phocidae have been found or recognized, either near the Central American Seaway, or on the east coast of North America, or in Europe. Hence, it can be hypothesized that direct ancestors of Phocidae or the earliest Phocidae crossed the North Atlantic Ocean from the Central American Seaway to the Mediterranean Sea during the late Oligocene or earliest Miocene, shortly after diversification of early Pinnipedimorpha and Pinnipedia in the northeast Pacific. Direct migration from the Central American Seaway to the Mediterranean may indeed explain (1) the record of a monachine mandible in the Burdigalian to Langhian of Libya (*Afrophoca libyca*) [36], (2) the early diversification of Phocidae taking place in Europe [9,10], and (3) the lower diversity of the Phocidae during the early and middle Miocene along the east coast of North America, as compared to Europe [10].

## 7. Conclusion

Formerly, the extinct phocid genus *Monotherium* included five different species: *Monotherium aberratum* Van Beneden, 1876; *Monotherium affine* Van Beneden, 1876; *Monotherium delognii* Van Beneden, 1876; *Monotherium gaudini* (Guiscardi, 1870); and *Monotherium*? *wymani* (Leidy, 1853). After a careful revision, only the type species *M. delognii* is retained, and considered Phocidae indet., sharing affinities with the late middle to late Miocene North Sea stem phocine species *Prophoca rousseaui*. *Monotherium aberratum* and *M. affine* are replaced into the new, most likely phocine, genus *Frisiphoca*. Most of the original material from the late Miocene of the North Sea is considered non-diagnostic, with only a lectotype humerus retained for *F. aberratum*, one lectotype humerus for *F. affine*, and one lectotype pelvis for *M. delognii*. *Frisiphoca aberratum*, *F. affine* and *M. delognii* are dated to the late Miocene, based on stratigraphic provenance in the literature.

First identified as *Phoca wymani* and being restricted here to its holotype ear region, the species *Monotherium*? *wymani* from the east coast of North America cannot be compared to *M. delognii*, but the type tympanic bullae could be considered diagnostic; the taxon is not treated in more detail, pending the discovery of more complete fossils. However, some specimens previously referred to *Monotherium* from the east coast of North America share affinities with *F. aberratum* and *F. affine* from the North Sea Basin, indicating a certain degree of faunal communication across the North Atlantic during the late Miocene.

Finally, the Italian species *Monotherium gaudini* is replaced into a new genus within the subfamily Monachinae (as confirmed with the phylogenetic analysis): *Noriphoca gaudini*. A careful literature study of *N. gaudini* reveals the taxon as one of the two oldest, or even the oldest known phocid, dated to the Chattian (late Oligocene) or Aquitanian (early Miocene). This is also the oldest known record of a crown pinniped, replacing the desmatophocid *Desmatophoca brachycephala* from the Aquitanian of the northeast Pacific as the oldest known crown pinniped. Previously, Dewaele *et al*. [10] questioned the validity of published phocid fossil records from the Oligocene of South Carolina, USA [30] and the Eocene of Germany [31]. The revised stratigraphic origin of *N. gaudini* pushes the origination and the migration of the direct ancestors of Phocidae or the earliest Phocidae through the Central American Seaway and eastwards across the Atlantic back to the late Oligocene and indicates a very early diversification of Pinnipedimorpha into the different families of Pinnipedia.

## Supplementary Material

Supplemental information 1: measurements, biostratigraphic and phylogenetic data.

## Supplementary Material

Supplemental information 2: phylogenetic character matrix

## Supplementary Material

Supplemental information 3: reassigned Monotherium specimens.

## Data accessibility

All data for this paper are available within this paper or as electronic supplementary material of this paper. Length measurements of specimens, dinoflagellate cyst biostratigraphy results, and the phylogenetic matrix are provided as electronic supplementary material. This published work and the nomenclatural acts it contains have been registered in ZooBank. The LSID for this publication is urn:lsid:zoobank.org:pub:17B81201-199E-4577-B465-79F65A108A8.
