# Supplementary material for: A critical revision of the fossil record, stratigraphy and diversity of the Neogene seal genus Monotherium (Carnivora, Phocidae)
Source: R Soc Open Sci. 2018 May 9;5(5):171669. doi: 10.1098/rsos.171669 (PMC5990722; doi:10.1098/rsos.171669)
Supplement: Supplemental information 1: measurements, biostratigraphic and phylogenetic data. [file rsos171669supp1.docx]

**Supplementary Information 1**

List of supplemental tables and figures:

**1. Measurements**

Supplemental table 1: Measurements of vertebrae.

Supplemental table 2: Measurements of astragalus.

Supplemental table 3: Measurement of calcaneum.

**2. Comparative taxa**

Supplemental list 1: Extant taxa.

Supplemental list 2: Fossil taxa.

**3. Dinoflagellate cyst biostratigraphy**

Supplemental table 4: List of dinoflagellate cyst species and acritarchs.

**4. Phylogeny**

Supplemental list 3: Character list for the phylogenetic analysis.

Supplemental figure 1: Node numbers on the 50% bootstrap consensus tree for the apomorphy list of Supplemental table 7.

Supplemental table 5: List of apomorphies and character changes between nodes within the 50% bootstrap consensus tree.

**5. References**

**1. Measurements**

Measurements of the lectotype humeri of *Frisphoca aberratum* and *Frisiphoca affine*, as well as other specimens from the Neogene of Belgium that have formerly been assigned to *Monotherium,* have been taken with a mechanical caliper, with a resolution of 0.1 mm. For reasons of consistency, measurements of the humerus and presented here follow the scheme used by [1] and a number of subsequent publications [2, 3]; but note that not all characters observed by these authors have been retained in the present study. When erosion is limited, ‘+’ indicates minimum value measured due to erosion and actual values should be –only slightly– larger. When structures are impossible to measure, either due to fractures or progressed erosion, ‘N/A’ is used.

**Supplemental table 1:** Measurements of vertebrae (in mm).

|  | IRSNB 1108-M255a (*Monotherium delognii*; Phocidae indet.) | IRSNB 1108-M255b (*Monotherium delognii*; Phocidae indet.) | IRSNB 1217-M256a (*Monotherium delognii*; Phocidae indet.) (caudal) | IRSNB M1217-M256b (*Monotherium delognii*) (caudal) | IRSNB M1219 (*Monotherium aberratum*) (lumbar) | IRSNB M1190 (*Monotheriumf affine*) (lumbar) | IRSNB M1114 (*Monotherium affine*) (lumbar) | IRSNB 1187-M273a (*Monotherium aberratum*) (first caudal) | IRSNB 1187-M273b (*Monotherium aberratum*) (second caudal) | IRSNB 1187-M273c (*Monotherium aberratum*) (third caudal) |
| --- | --- | --- | --- | --- | --- | --- | --- | --- | --- | --- |
| Absolute height | N/A | N/A | 35.5 | +32.7 | N/A | N/A | N/A | 20.7 | 16.4 | 15.5 |
| Width across transverse processes | N/A | N/A | N/A | 43.2 | N/A | N/A | N/A | 28.6 | 27.1 | N/A |
| Height body | +44.3 | +52.0 | 26.1 | +23.6 | N/A | N/A | N/A | 14.8 | 15.1 | 13.3 |
| Width body | +60.6 | +57.0 | 27.0 | 26.5 | +38.2 | 47.2 | 56.8 | 15.7 | 15.2 | 13.3 |
| Length body | 95.8 | N/A | 42.8 | 47.2 | 55.5 | N/A | 73.8 | 26.1 | 26.2 | 25.1 |

**Supplemental table 2:** Measurements of astragalus IRSNB 1144-M272 of indeterminate Phocidae (in mm).

|  | Phocidae indet. |
| --- | --- |
|  | IRSNB 1144-M272 |
| Absolute length | c. 56.2 |
| Maximum dorsoplantar height | N/A |
| Mediolateral width across tibial facet | 18.1 |
| Dorsoplantar height astragalar head | N/A |
| Mediolateral width astragalar head | N/A |
| Dorsoplantar height caudal process | N/A |
| Mediolateral width caudal process | 18.5 |
| Maximal length ectal facet | 19.4 |
| Maximal length sustentacular facet | N/A |

**Supplemental Table 3:** Measurement of calcanea IRSNB 1187-M273d and IRSNB M1144 of indeterminate Phocidae (in mm).

|  | Monachinae indet.. | |
| --- | --- | --- |
|  | IRSNB 1187-M273d | IRSNB M1144 |
| Absolute proximodistal length | 51.2 | 56.8 |
| Maximal mediolateral width | 25.1 | 27.6 |
| Least mediolateral width of calcaneal tuber | 11.1 | 13.1 |
| Mediolateral width across the medial calcaneal tuberosity | 16.7 | 15.3 |
| Maximal dorsoplantar height | 24.2 | 30.2 |
| Maximal length of ectal facet | 14.5 | 23.1 |
| Heigth of the ectal facet | 5.5 | N/A |
| Maximal length of sustentacular facet | 22.8 | 22.6 |
| Mediolateral width of facet for navicular | 11.7 | 13.1 |
| Dorsoplantar height of facet for navicular | 14.9 | N/A |

**2. Comparative material**

**Supplemental list 1:** Extant species and specimens used as comparative material for the re-investigation of *Monotherium*.

***Comparative Material (extant taxa)—****Cystophora cristata* (Erxleben, 1777) (USNM 118962, USNM 550411), *Erignathus barbatus* Erxleben, 1777 (USNM 230952, USNM 300704; USNM 500250, USNM 500251), *Halichoerus grypus* (Fabricius, 1791) (IRSNB 12550, IRSNB 34548, USNM 53291), *Histriophoca fasciata* (Zimmermann, 1783) (USNM 399449, USNM 504959, USNM 504960, USNM 571367), *Hydrurga leptonyx* (Blainville, 1820) (IRSNB 15388), *Leptonychotes weddellii* (Lesson, 1826) (IRSNB 15390), *Lobodon carcinophaga* (Hombron & Jacquinot, 1842) (IRSNB 13307), *Monachus monachus* Hermann, 1779 (IRSNB 1153), *Ommatophoca rossi* (Gray, 1844) (IRSNB 1164), *Pagophilus groenlandicus* (Erxleben, 1777) (IRSNB 1555D), *Phoca vitulina* (IRSNB 39043), *Pusa hispida* (USNM 225778, USNM 341617), *Pusa caspica* (Gmelin, 1788) (USNM 341615, USNM 341616), *Pusa sibirica* (Gmelin, 1788) (IRSNB 15264, IRSNB 21171).

**Supplemental list 2:** Extinct species and specimens used as comparative material for the re-investigation of *Monotherium*.

***Comparison Material (extinct taxa)—***Not considering the “*Phoca*” (*Nanophoca*) *vitulinoides* specimens, which are listed in the ‘referred specimens’ section below and Supplemental Information. *Acrophoca longirostris* (holotype MNHN.F.SAS 563), *Batavipusa neerlandica* Koretsky & Peters, 2008 (MAB 3798, MAB 04342), *Cryptophoca maeotica* (Nordmann, 1860) (USNM 489174 cast, USNM 489179 cast), *Leptophoca proxima* (lectotype IRSNB 1146-M279, IRSNB 1145-M280a-b, USNM 5359, USNM 5361, USNM 23224, USNM 23243, USNM 23450, USNM 175578, USNM 186990, USNM 205499, USNM 263648, USNM 284721, USNM 305247 cast, USNM 321934, USNM 411889, USNM 412115, USNM 454770), *Monachopsis pontica* (Eichwald, 1850) (USNM 1802 cast, USNM 214967 cast), *Piscophoca* *pacifica* (holotype MNHN.F.SAS 564, MNHN.F.SAS 488, MNHN.F.SAS 682). *Praepusa vindobonensis* (USNM cast of humerus without number, cast USNM 214964, cast USNM 214993; original specimens figured in Koretsky, 2001), *Prophoca rousseaui* Van Beneden, 1877 (lectotype IRSNB 1147-M275, IRSNB 1149-M274, IRSNB1150-M277a-b, IRSNB 1192-M276a-d IRSNB M2234, IRSNB-VERT-3250-15), *Sarmatonectes sintsovi* Koretsky, 2001 (cast USNM 1713/146, USNM cast of femur without number).

**3. Dinoflagellate cyst biostratigraphy**

**Supplemental table 4:** Quantitative list of the dinoflagellate cyst species and acritarchs recorded in the samples 1108LDW-1100Lab and 1132LDW-1102Lab. Bold and in italics: species with biostratigraphic significance.

| **Dinoflagellate cysts** | 1108LDW-1100Lab | 1132LDW-1102Lab |
| --- | --- | --- |
| *Achomosphaera andalousiensis* | ***1*** |  |
| *Apteodinium tectatum* |  | 1 |
| *Barssidinium wrennii* |  | ***1*** |
| *Baticasphaera minuta* | 1 |  |
| *Cerebrocysta* sp. indet. | 1 |  |
| *Habibacysta tectata* |  | ***1*** |
| *Lejeunecysta mariae* | 1 |  |
| *Lejeunecysta* sp. indet. |  | 1 |
| *Lingulodinium machaerophorum* | 3 | 8 |
| *Operculodinium centrocarpum* | 1 | 2 |
| *Operculodinium*? *eirikianum* |  | ***1*** |
| *Operculodinium israelianum* | 13 |  |
| *Operculodinium tegillatum* |  | ***2*** |
| *Operculodinium* sp. indet. | 1 |  |
| *Quinquecuspis concreta* |  | ***1*** |
| *Selenopemphix brevispinosa* | ***1*** | ***1*** |
| *Spiniferites* sp. indet. | ***1*** | ***4*** |
|  |  |  |
| **Acritarchs** |  |  |
| *Paralecaniella indentata* |  | 4 |
| Small spiny acritarchs |  | 1 |
| Reworked (*Wetzeliella* sp. indet.) | 2 |  |

**4. Phylogeny**

**Supplemental list 3:** Character list for the phylogenetic analysis, including references to selected recent publications from which the characters have been taken, or adapted. Note that these references refer either to characters presented for the first time by these authors, or to characters adopted or adapted from other sources: see references therein.

**Cranial, mandibular, and dental characters (unordered unless stated otherwise).**

1. Premaxilla-nasal suture: (0) extensive; (1) weakly reduced; (2) strongly reduced. [4-8]
2. Premaxilla-maxilla suture: 0) entirely lateral to nasal cavity; 1) anterior portion partially included in nasal cavity; (2) entirely within nasal cavity. [4-8] Adjusted from other studies because the additional condition (2) has been observed in *Cystophora cristata* and *Mirounga*.
3. Nasals, shape of posterior edge: (0) pointed; (1) rounded or square; (2) frontals insert between nasals. [4-7]
4. Facial angle: (0) snout more anteriorly than dorsally (angle 45° or more); (1) snout opening more dorsally than anteriorly (angle 45° or less). [5, 7]
5. Lateral border of opening of nasal cavity in lateral view: (0) rectilinear or weakly concave; (1) strongly concave. [7]
6. Position of posterior end of nasals: (0) anterior to maxilla-frontal suture; (1) posterior to maxilla-frontal suture but greatly anterior to the level of the jugal-squamosal suture; (2) almost reaches the level of the jugal-squamosal suture. [4, 7]
7. Alveolar process of maxilla: (0) facing ventrally; (1) facing anteroventrally posterior to P1. [7]
8. Maxillary process of jugal (at level of the anteroventral border of the orbit) in lateral view: (0) thin and low and increasing progressively posteriorly; (1) thick and high and increasing abruptly posteriorly. [7]
9. Position of anterior opening of infraorbital foramen in ventral view: (0) anterior to M1; (1) level or posterior to M1. [7]
10. Jugal, anterior end in dorsal view: (0) lateral to infraorbital foramen; (1) above or medial to the lateral margin of the infraorbital foramen. [7]
11. Jugal, direction of arch of anterior portion: (0) downwards; (1) flat, no distinct arch; (2) upwards. [5, 7]
12. Squamosal-jugal articulation: (0) splintlike; (1) mortised. [4, 5, 7]
13. Ventral edge of the zygomatic arch, in anterior view: (0) higher than alveolar plane; (1) level with the alveolar plane (or very close to). [5, 7]
14. Supraorbital process of frontal: (0) absent or weakly developed; (1) strongly developed. [4, 5, 7]
15. Orbital vacuities: (0) absent; (1) present. [4, 5]
16. Interorbital, least width: 0) in posteriormost portion of interorbital septum; 1) in anterior half of the interorbital septum. [4-7]
17. Interorbital least width:braincase width ratio: (0) high (i.e., much more than 20% of width of skull); (1) moderate (i.e., approximately 20%); (2) low (i.e., much less than 20%); (3) very low (i.e., 5% or less). [1, 3, 7, 9]
18. Major axes of glenoid fossae: (0) sub-parallel; (1) slightly convergent posteriorly. [7]
19. Orientation of medial margins of tympanic bullae: (0) diverging posteriorly; (1) parasagittal. [7]
20. Lateral end of tympanic bulla: (0) medial to level of mid-width of glenoid fossa; (1) lateral to level of mid-width of glenoid fossa. [7]
21. Inflation of tympanic bulla: (0) weak; (1) moderate; (2) strong. [1, 3-5, 7]
22. Carotid canal, posterior opening: (0) visible in ventral view (i.e., at least partially facing ventrally); (1) not visible in ventral view (i.e., opening having very little ventral aspect). [4-7]
23. Posterior opening of the carotid canal and posterior lacerate foramen: (0) clearly separated; (1) coalescent. [7]
24. Mastoid: 0) not visible in dorsal view; 1) visible. [4-7]
25. Heavily pachyosteosclerotic mastoid: (0) absent; (1) present [4, 5, 7]
26. Relation of paroccipital process to mastoid: (0) connected by a low and discontinuous ridge; (1) connected by a high and continuous ridge; (2) well separated. [1, 3-5]
27. Pterygoid process: (0) rounded with convex lateral margin; (1) flat with concave lateral margin. [4, 7]
28. Alisphenoid canal: (0) present; (1) absent. [4, 5, 7]
29. Direction of occipital condyles in occipital view: (0) ventral; (1) diverging dorsally. [7]
30. Shape of head of malleus: (0) broad and circular; (1) slender and elliptic. [7]
31. Tooth rows: (0) parallel; (1) diverging posteriorly [5, 7]
32. Upper incisors: (0) three; (1) two; (2) one (ordered). [1, 3-9]
33. Premolars: (0) parallel to tooth row axis; (1) obliquely oriented in tooth row. [1, 3, 5, 9]
34. Diastema between P4 and M1: (0) large; (1) reduced. [5]
35. Lower incisors: (0) three; (1) two; (2) one (ordered). [1, 3-5, 7-9]
36. Lateral incisor, relative size: (0) incisiform; (1) intermediate shape; (2) caniniform. [1, 3-5, 8, 9]
37. Upper incisor, roots: (0) strongly transversely compressed; (1) moderately transversely compressed. [4-6, 8]
38. P2-4, p2-4, roots: (0) double-rooted; (1) single-rooted. [1, 3-5, 7, 9]
39. Postcanine teeth, crowns: (0) multi-cusped; (1) single-cusped. [1, 3-5, 7-9]
40. Postcanine teeth, lingual cingulum: (0) well developed; (1) not or poorly developed. [1, 3-5, 7-9]
41. M1 and m1, roots: 0) double-rooted; 1) single-rooted. [4, 5, 7]
42. M2: (0) present; (1) absent. [4, 5, 7]
43. p4, size comparison m1: (0) about equal in size; (1) P4 and p4 larger than M1 and m1. [1, 3-5, 8, 9]]

**Postcranial characters (all are unordered).**

1. Atlas, transverse foramen: (0) visible in posterior view; (1) at least partially visible in dorsal view. [4, 7]
2. Atlas, direction of transverse process in lateral view: (0) oblique; (1) sub-vertical. [7]
3. Scapula: (0) two ridges on lateral side do not join near glenoid; (1) two ridges on lateral side join near glenoid (this study, see Hodgetts [10])
4. Humerus, lesser tubercle and head: (0) head higher or at same level as lesser tubercle; (1) tubercle higher. [1, 3, 4, 7-9]
5. Humerus, greater tubercle height: (0) below level of head; (1) at level of head or slightly above; (2) above level of head. [1, 3, 4, 6-9]
6. Humerus, supinator: (0) strongly developed; (1) poorly developed. [4-8]
7. Humerus, deltopectoral crest; (0) smooth distal termination; (1) sharp distal termination. [4, 5]
8. Humerus, length of deltoid crest: (0) shorter than or subequal to one-half length of the bone, confined to the proximal half of the bone; (1) longer than one-half length of the bone. [1, 3-5, 7-9]
9. Humerus, transverse bar in bicipital groove: (0) absent; (1) present. (this study)
10. Humerus, entepicondylar foramen: (0) absent; (1) present. [4-8]
11. Radius, location radial tuberosity: 0) medial side; 1) posteromedial side. [7]
12. Ulna, distal end of styloid process: 0) distally pointed; 1) flattened. [7]
13. Metacarpal I, length: (0) slightly longer than mcII; (1) much longer. [5, 6, 8]
14. Metapodials, head: (0) keeled with trochleated phalangeal articulations; (1) smooth, with phalanges flat, articulations hingelike. [4, 6-8]
15. Sacrum, number of fused vertebrae: (0) three; (1) four. (this study)
16. Innominate, anterodorsal iliac spine: (0) dorsal to anteroventral iliac spine; (1) posterodorsal to the anteroventral iliac spine. [7]
17. Innominate, posteroventral iliac spine (=iliac tuberosity): (0) small or absent; (1) large and strongly protruding. [7]
18. Innominate, iliopectineal eminence: (0) strongly developed; (1) moderately well developed; (2) small or absent. (this study)
19. Innominate, ilium: (0) shallow gluteal fossa; (1) deep gluteal fossa. [5-7]
20. Innominate, ilium: (0) weakly everted wing; (1) moderately everted; (2) strongly everted wing. [5-7]
21. Innominate, ilium: (0) long, compared to postacetabular region; (1) short. [4-7]
22. Innominate, ischial spine: (0) unenlarged; (1) enlarged. [4]
23. Femur, lesser trochanter: (0) present; (1) absent. [1, 3-6, 9]
24. Femur, neck, relative size: (0) thick; (1) narrow. [1, 3, 8, 9]
25. Femur, distal condyles: (0) roughly similar in size or slight size difference; (1) large size difference. [1, 3, 8, 9]]
26. Femur, epiphyses: (0) distal epiphysis wider than proximal; (1) widths of proximal and distal epiphyses about equal; (2) proximal epiphysis wider than distal one. [1, 3, 8, 9]
27. Femur, diaphysis: (0) minimum width less than or about equal to two-thirds width of proximal epiphysis; (1) minimum width more than two-thirds width of proximal epiphysis. [1, 3, 9]
28. Femur, head and greater trochanter: (0) head reaches higher than greater trochanter; (1) both reach same level; (2) greater trochanter reaches higher than head. [1, 3-6, 8, 9]
29. Femur, trochanteric fossa: (0) little reduced; (1) strongly reduced or absent. [4, 5, 8]
30. Femur, orientation of fossa for *m. peroneus longus*: (0) lateral; (1) anterolateral. [7]
31. Tibia and fibula: (0) proximal epiphyses not fused; (1) proximal epiphyses fused. (this study)
32. Tibia, development post-tibial fossa: (0) weak; (1) strong. [3-6]
33. Astragalus, calcaneal process: (0) absent; (1) poorly developed; (2) well developed. [4, 7]
34. Sustentacular facet of the astragalus: (0) oval-shaped and narrowed at contact with cuboid facet; (1) long (at least twice longer than wide), slender and strongly bent medially; (2) short and tongue-like with no narrowing at contact with cuboid facet. [7, 8]
35. Calcaneum, articular surface for fibula: (0) absent or very reduced; (1) well developed. [7]
36. Metatarsal I, articular surface for metatarsal II: (0) oriented laterally; (1) oriented dorsolaterally; (2) inconspicuous. [7]
37. Metatarsal III, length: (0) less than 50% shorter than metatarsal I; (1) approximately 50% shorter (or more) than metatarsal I. [7]

**
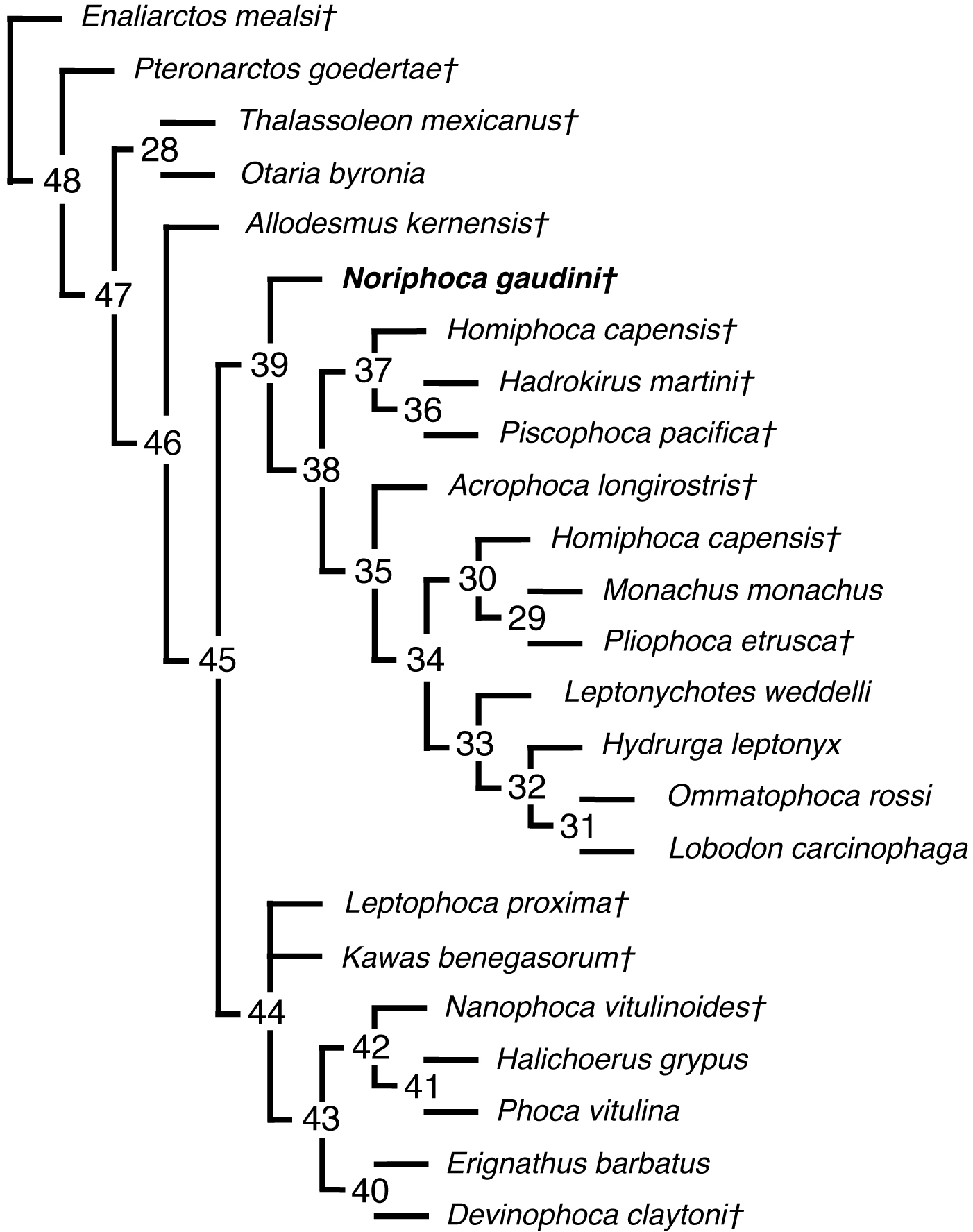
**

**Supplemental figure 1:** Node numbers on the most parsimonous phylogenetic tree represent the node numbers as used to identify (syn)apomorphies in Supplemental Table 6.

**Supplemental table 5:** List of apomorphies and character changes between nodes within the most parsimonous phylogenetic tree. The list of apomorphies results from the analysis using PAUP and as explained in the study. Ambiguous (syn)apomorphies are indicated by a simple arrow (-->), and unambiguous (syn)apomorphies by a double arrow (==>). Node numbers are specified on the tree above (Supplemental figure 1).

| **Branch** | **Character number** | **Consistency Index** | **State change** |
| --- | --- | --- | --- |
| *Enaliarctos mealsi* <–> node 48 | 17  18  26  34  35  48  49  57  61  67  69  72 | 0.286  0.500  1.000  0.167  1.000  0.286  0.500  0.500  0.667  0.167  0.400  0.200 | 1 <=> 0  0 <=> 1  1 <=> 0  0 <=> 1  1 <–> 0  1 <–> 0  1 <–> 0  1 <–> 0  0 <–> 2  1 <–> 0  0 <–> 2  1 <–> 0 |
| Node 48 ––> *Pteronarctos goedertae* | 20  30  36  43 | 0.500  0.333  0.400  0.333 | 0 ==> 1  0 ––> 1  0 ==> 1  1 ==> 0 |
| Node 48 ––> node 47 | 3  4  8  15  31  38 | 0.667  0.250  0.333  1.000  0.500  0.400 | 1 ––> 0  0 ––> 1  1 ==> 0  0 ==> 1  1 ––> 0  0 ==> 1 |
| Node 47 ––> node 28 | 3  14  23  48  54  79 | 0.667  1.000  1.000  0.286  0.333  1.000 | 0 ––> 2  0 ==> 1  0 ––> 1  1 ==> 2  1 ==> 0  0 ––> 2 |
| Node 28 ––> *Thalassoleon mexicanus* | 10  43  50  51 | 0.250  0.333  0.333  0.333 | 0 ==> 1  1 ==> 0  0 ==> 1  1 ==> 0 |
| Node 28 ––> *Otaria flavescens* | 4  6  11  34  36  38  39  41  69  74 | 0.250  0.667  0.286  0.167  0.400  0.400  0.167  0.250  0.400  0.500 | 1 ––> 0  1 ==> 0  0 ==> 2  0 ==> 1  0 ==> 2  1 ––> 2  0 ==> 1  0 ==> 1  0 ––> 1  0 ==> 1 |
| Node 47 ––> node 46 | 1  2  9  12  17  26  27  40  66  68  70  76 | 0.500  0.500  0.500  1.000  0.286  1.000  1.000  0.250  0.500  0.333  0.250  1.000 | 0 ––> 1  0 ––> 1  0 ==> 1  0 ==> 1  1 ==> 2  1 ==> 2  0 ==> 1  1 ––> 0  0 ==> 1  0 ==> 1  0 ––> 1  0 ==> 1 |
| Node 46 ––> *Allodesmus kernensis* | 6  38  39  41  48  50  69 | 0.667  0.400  0.167  0.250  0.286  0.333  0.400 | 1 ==> 2  1 ––> 2  0 ==> 1  0 ==> 1  1 ––> 0  0 ==> 1  0 ––> 2 |
| Node 46 ––> node 45 | 1  11  20  21  25  28  29  31  42  44  55  64  65  67  72  74  76  77 | 0.500  0.286  0.500  1.000  1.000  1.000  0.500  0.500  1.000  0.250  0.500  1.000  1.000  0.167  0.200  0.500  1.000  0.667 | 1 ––> 2  0 ––> 1  0 ==> 1  0 ––> 1  0 ==> 1  0 ==> 1  0 ==> 1  0 ––> 1  0 ==> 1  0 ––> 1  0 ––> 1  0 ==> 1  0 ==> 1  1 ––> 0  1 ––> 0  0 ==> 1  1 ==> 2  0 ==> 1 |
| Node 45 ––> node 39 | 10  13  19  43  54  59  73  78  79  80 | 0.250  0.333  0.333  0.333  0.333  1.000  0.500  0.500  1.000  1.000 | 0 ==> 1  0 ==> 1  0 ––> 1  1 ––> 0  1 ––> 0  1 ––> 0  0 ––> 1  0 ––> 1  0 ––> 1  0 ––> 1 |
| Node 39 ––> node 38 | 5  32  39 | 0.333  1.000  0.167 | 0 ––> 1  0 ==> 1  0 ==> 1 |
| Node 38 ––> node 35 | 36  56  68  72  77 | 0.400  0.500  0.333  0.200  1.000 | 0 ==> 1  0 ––> 1  1 ==> 0  0 ––> 1  1 ==> 2 |
| Node 35 ––> *Acrophoca longirostris* | 5  37 | 0.333  0.500 | 1 ––> 0  1 ==> 0 |
| Node 35 ––> node 34 | 11  17  34  45  61  70 | 0.286  0.286  0.167  0.500  0.667  0.250 | 1 ––> 0  2 ––> 1  0 ––> 1  0 ==> 1  0 ==> 1  1 ––> 0 |
| Node 34 ––> node 30 | 13  19  78 | 0.333  0.333  0.500 | 1 ==> 0  1 ==> 0  1 ==> 0 |
| Node 30 ––> node 29 | 33  44  52  67 | 0.333  0.250  0.333  0.167 | 0 ==> 1  1 ––> 0  0 ==> 1  0 ==> 1 |
| Node 29 ––> *Pliophoca etrusca* | 47  68  73 | 0.200  0.333  0.500 | 0 ==> 1  0 ==> 1  1 ==> 0 |
| Node 29 ––> *Monachus monachus* | 56 | 0.500 | 1 ==> 0 |
| Node 38 ––> *Mirounga leonina* | 2  11  29  35  38  41  48 | 0.500  0.286  0.500  1.000  0.400  0.250  0.286 | 1 ==> 2  0 ––> 1  1 ==> 0  1 ==> 2  1 ==> 2  0 ==> 1  1 ==> 0 |
| Node 34 ––> node 33 | 6  9  1 | 0.667  0.500  0.250 | 1 ==> 2  1 ==> 0  1 ==> 0 |
| Node 33 ––> *Leptonychotes weddelli* | 17  34 | 0.286  0.167 | 1 ––> 2  1 ––> 0 |
| Node 33 ––> node 32 | 39  40  47 | 0.167  0.250  0.200 | 1 ––> 0  0 ==> 1  0 ==> 1 |
| Node 32 ––> node 31 | 17  48  52  70 | 0.286  0.286  0.333  0.250 | 1 ––> 0  1 ==> 0  0 ==> 1  0 ==> 1 |
| Node 31 ––> *Lobodon carcinophaga* | 30  44  71  72 | 0.333  0.250  0.500  0.200 | 0 ==> 1  1 ==> 0  0 ==> 1  1 ==> 0 |
| Node 31 ––> *Ommatophoca rossi* | 2  7  24  39 | 0.500  0.500  0.500  0.167 | 1 ==> 2  0 ==> 1  0 ==> 1  0 ––> 1 |
| Node 32 ––> *Hydrurga leptonyx* | 51 | 0.333 | 1 ==> 0 |
| Node 38 ––> node 37 | 1  11  30  33 | 0.500  0.286  0.333  0.333 | 2 ––> 1  1 ––> 2  0 ==> 1  0 ––> 1 |
| Node 37 ––> 36 | 7  8  13  52 | 0.500  0.333  0.333  0.333 | 0 ==> 1  0 ==> 1  1 ==> 0  0 ––> 1 |
| Node 36 ––> *Hadrokirus martini* | 11  34 | 0.286  0.167 | 2 ––> 1  0 ==> 1 |
| Node 36 ––> *Piscophoca pacifica* | 1  33  45 | 0.500  0.333  0.500 | 1 ––> 2  1 ––> 0  0 ==> 1 |
| Node 37 ––> *Homiphoca capensis* | 17  47  53  67  75 | 0.286  0.200  0.500  0.167  0.500 | 2 ==> 0  0 ==> 1  0 ==> 1  0 ==> 1  0 ==> 1 |
| Node 45 ––> node 44 | 2  4  18  21  22  24  40  49  53  57  58  60  61  70  71  75 | 0.500  0.250  0.500  1.000  1.000  0.500  0.250  0.500  0.500  0.500  1.000  1.000  0.667  0.250  0.500  0.500 | 1 ––> 0  1 ––> 0  0 ––> 1  1 ––> 2  0 ––> 1  0 ––> 1  0 ––> 1  1 ==> 0  0 ==> 1  1 ==> 0  1 ––> 2  0 ==> 1  0 ==> 1  1 ––> 0  0 ==> 1  0 ==> 1 |
| Node 44 ––> node 43 | 40  47  48  50  51  63  69 | 0.250  0.200  0.286  0.333  0.333  1.000  0.400 | 1 ––> 0  0 ––> 1  1 ––> 0  0 ==> 1  1 ==> 0  0 ==> 1  0 ––> 1 |
| Node 43 ––> node 40 | 3  5  48  72 | 0.667  0.333  0.286  0.200 | 0 ==> 1  0 ==> 1  0 ––> 2  0 ––> 1 |
| Node 42 ––> *Erignathus barbatus* | 17 | 0.286 | 2 ==> 1 |
| Node 42 ––> *Devinophoca claytoni* | 19  34  36  40 | 0.333  0.167  0.400  0.250 | 0 ==> 1  0 ==> 1  0 ==> 1  1 ––> 0 |
| Node 43 ––> node 42 | 11  16  37  46  55  62 | 0.286  1.000  0.500  1.000  0.500  0.500 | 1 ––> 2  0 ––> 1  1 ––> 0  0 ==> 1  1 ––> 0  0 ––> 1 |
| Node 42 ––> node 41 | 63 | 1.000 | 1 ==> 2 |
| Node 43 ––> *Halichoerus grypus* | 4  8  36  38  39  41 | 0.250  0.333  0.400  0.400  0.167  0.250 | 0 ––> 1  0 ==> 1  0 ==> 2  1 ==> 2  0 ==> 1  0 ==> 1 |
| Node 41 ––> *Phoca vitulina* | 10 | 0.250 | 0 ==> 1 |
| Node 42 ––> *Nanophoca vitulinoides* | 47  67  69  71 | 0.200  0.167  0.400  0.500 | 1 ––> 0  0 ==> 1  1 ––> 0  1 ==> 2 |
| Node 44 ––> *Kawas benegasorum* | 62  71 | 0.500  0.500 | 0 ==> 1  1 ==> 2 |
| Node 44 ––> *Leptophoca proxima* | 66  67 | 0.500  0.167 | 1 ==> 0  0 ==> 1 |

**5. References**

1. Koretsky IA. 2001 Morphology and systematics of the Miocene Phocinae (Mammalia: Carnivora) from Paratethys and the North Atlantic Region. *Geologica Hungarica series Palaeontologica* **54**, 1–109.
2. Koretsky IA, Ray CE Peters N. 2012 A new species of *Leptophoca* (Carnivora, Phocidae, Phocinae) from both sides of the North Atlantic Ocean (Miocene seals of the Netherlands, part I). *Deinsea* **15**,1-12.
3. Koretsky IA, Rahmat SJ. 2013. First record of fossil Cystophorinae (Carnivora, Phocidae): middle Miocene seals from the northern Paratethys. *Rivista Italiana di Paleontologia e Stratigrafia* **119**, 325-350.
4. Berta A, Wyss AR. 1994 Pinniped phylogeny. *Proceedings of the San Diego Society of Natural History* **29**, 33-56.
5. Bininda-Emonds ORP, Russell AP. 1996 A morphological perspective on the phylogenetic relationships of the extant phocid seals (Mammalia: Carnivora: Phocidae). *Bonner Zoologische Monographien* **41**, 1-256.
6. Cozzuol MA. 2001 A ‘Northern” seal from the Miocene of Argentina: Implications for phocid phylogeny and biogeography. *Journal of Vertebrate Paleontology* **21**, 415-421. (doi:10.1671/0272-4634(2001)021[0415:ANSFTM]2.0.C0;2)
7. Amson E, Muizon C de. 2014 A new durophagous phocid (Mammalia: Carnivora) from the late Neogene of Peru and considerations on monachine seal phylogeny. *Journal of Systematic Paleontology* **12**, 523-548.
8. Berta A, Kienle S, Bianucci G, Sorbi S. 2015 A Reevaluation of *Pliophoca etrusca* (Pinnipedia, Phocidae) from the Pliocene of Italy: Phylogenetic and Biogeographic Implications. *Journal of Vertebrate Paleontology* **35**, e889144. (doi:10.1080/02724634.2014.889144)
9. Koretsky IA, Grigorescu D. 2002 The Fossil Monk Seal Pontophoca sarmatica (Alekseev) (Mammalia: Phocidae: Monachinae) from the Miocene of Eastern Europe. Pp. 149–162 in R. J. Emry (ed.), Cenozoic Mammals of Land and Sea: Tributes to the Career of Clayton E. Ray. *Smithsonian Contributions to Paleobiology* **93**.
10. Hodgetts LM. 1999 Animal bones and human society in the late younger stone age of arcticNorway; volume 2 of 2: figures and appendices. PhD thesis, University of Durham.
