# Supplementary material for: A critical revision of the fossil record, stratigraphy and diversity of the Neogene seal genus Monotherium (Carnivora, Phocidae)
Source: R Soc Open Sci. 2018 May 9;5(5):171669. doi: 10.1098/rsos.171669 (PMC5990722; doi:10.1098/rsos.171669)
Supplement: Supplemental information 3: reassigned Monotherium specimens. [file rsos171669supp3.docx]

**Revision of *Monotherium*: Supplementary information 3**

This supplementary file provides the reassessment and illustration of additional specimens that have formerly been identified as *Monotherium* from Belgium, but that are currently considered unidentifiable beyond the family or subfamily level [1]. This file includes specimens that Van Beneden [1] illustrated, as well as unfigured referred specimens. For specimens illustrated by Van Beneden [1], original plate and figure numbers are mentioned in the “referred specimens” sections. Additionally, specimens that have not been illustrated are not accompanied by labels in the IRSNB collections. Therefore, exact geographic and stratigraphic locations remain unknown, although Van Beneden [1] assigned a ‘Diestian’ age to all specimens of *Monotherium* from Belgium. However, it should be noted that Van Beneden used the name *Monatherium* in his 1877 publication, instead of *Monotherium.* Although he provide an etymological reasoning for the name *Monatherium*, the original name *Monotherium* is the only valid name according to the International Code on Zoological Nomenclature (see main text). Therefore, it should be noted that although Van Beneden [1] assigned the considered specimens to *Monatherium aberratum*, *Monatherium affine*, and *Monatherium delognii*, he technically assigned them to *Monotherium aberratum*, *Monotherium affine*, and *Monotherium delognii*. In this document, we retain the original, erroneous, name of *Monatherium*, which Van Beneden [1] used, although we explicitly state that *Monatherium* is a junior synonym to *Monotherium*.

**Family Phocidae Gray, 1821**

**Subfamily Monachinae indet.**

**Referred specimens. *Associated baculum, caudal vertebrae, and partial left and right pes.*** IRSNB 1187-M273a-o: three caudal vertebrae (a-c), right calcaneum (d), right cuboid (e), right navicular (f), right second metatarsal (g), right first metatarsal (h), left first metatarsal (i), right fifth metatarsal (j), left fourth metatarsal (k); right intermediate ?first phalanx (l); distal phalanx (m), left intermediate ?fifth phalanx (n); baculum (o), left navicular (?), two or three phalanges (?), 'Diestian', 2^nd^ section at Borgerhout, originally assigned to *Monatherium aberratum* by Van Beneden [1: plate 17, figures 15-29]. ***Associated left pes.*** IRSNB 1188-M270a-b: phalanx (a), left fifth metatarsal (b), 'Diestian', 3^rd^ section at Borgerhout, originally assigned to *Monatherium aberratum* by Van Beneden [1: plate 17, figures 10, 11]. ***Associated phalanges.*** IRSNB M1131, four partial phalanges, originally assigned to *Monatherium affine* [1]. ***Isolated calcaneum.*** IRSNB M1144, right calcaneum, originally assigned to *Monatherium aberratum* [1].

**Comments.** A detailed reassessment of the original ‘Diestian’ attribution of the original specimens of *Monatherium* from Belgium is provided in the main text and not in the supplementary information.

**Description and comparison**

**Baculum, caudal vertebrae, and partial left and right pes (IRSNB 1187-M273a-o)**

**Caudal vertebrae (Supplementary figure 1A-C).** Three successive caudal vertebrae are associated with the partial pes: IRSNB 1187-M273a-c. Although the vertebrae are complete, the sutures between the bodies and the epiphyses are still visible. Nevertheless, the specimen can be considered skeletally mature [2]. The transverse processes of the most anterior one of the vertebrae (Ca3) extend along the entire length of the vertebra. Laterally, however, they are short and end in a cylindrical structure. The anterior articular and mammillary processes are blunt. The neural process is small, as in Monachinae, while this process is generally well developed in Phocinae. Contrasting to Ca3, the other caudal vertebrae lack a neural arch. All processes on Ca4 and Ca5 are blunt and small, corresponding to their small size. Anterior on the lateral surface of the body, there is a transverse process; posterior on the dorsal surface, there are two widely spaced dorsal processes. Posterior on the ventral surface, there are two hemal processes. These hemal processes are more closely spaced than the dorsal processes; they are larger in Ca5 than in Ca4. The identification of these caudal vertebrae as Ca3 (a), Ca4 (b), and Ca5 (c) is based on comparison with extant phocids, where Ca3 is characterized by the presence of a neural arch, while such a neural arch is absent in Ca4 and Ca5.

**Baculum (Supplementary figure 2A, B).** The baculum IRSNB 1187-M273o indicates that this specimen is a male. Fusion of all epiphyses indicates that the specimen is skeletally mature [2]. The baculum is long and slender (132.7 mm), thickest just distal to the base and tapering gradually to the apex. The proximal half of the baculum is relatively straight, but its proximal half gradually curves dorsally. An urethral groove is present on the dorsal side of the apex and becomes rapidly very deep towards the distal tip of apex.

**Calcaneum (Supplementary figure 2C-E).** One right calcaneum (IRSNB 1187-M273d) is associated with the partial pes. Unlike the calcaneum of Phocinae, this calcaneum and calcaneum IRSNB 1125-M263 (originally *Monatherium affine*; Phocidae *cf. Frisiphoca affine* in this study) are distally much wider than they are proximally, in lateral view. The ratio of the plantardorsal height to the total length of the calcaneum is 0.490 (25.1 mm : 51.2 mm), which lies just within the range of Phocinae, based on Muizon [3: table 8]. Extant Lobodontini have a high ratio (>0.55), other extinct phocids and extant Monachini an intermediate ratio, and extant phocines a low ratio (<0.50). Because of the unclear distinction of many extinct phocid calcanea from both subfamilies based on this ratio, this value of 0.490 for IRSNB 1187-M273d is of little diagnostic value. The calcaneal tuber bears a prominent medial process (=medial calcaneal tuber) at its proximal end, rounding the caudal process of the astragalus, as in Phocidae *cf. Frisiphoca affine* specimen IRSNB 1125-M263. On the dorsal margin of the calcaneum, approximately in the middle, there is a teardrop-shaped, not strongly developed concave facet for the articulation with the fibula. Among Phocidae, only extant Lobodontini and *Homiphoca* have a well-developed fibular facet on the calcaneum.

Forming a longitudinal crest, the trochlear process extends across the dorsal surface of the calcaneum anterior to the ectal facet (=proximal astragalar facet), as in other Phocidae [4]. On the lateral margin of the calcaneum, the peroneal process, supporting the tendon of *m. peroneus longus* and *m. peroneus brevis*, is well developed, as in Monachinae [3]. This peroneal process is oriented posterodorsally-anteroventrally, but appears almost sub vertical. The lateral surface of this process is slightly concave to guide the tendon of *m. peroneus longus* [3]. A deep and narrow troclear sulcus runs between the peroneal process and the trochlear process. This sulcus guides the tendon of *m. peroneus brevis*. The sustentaculum is small, and the secondary shelf of the sustentaculum is slender but prominently projecting medially, and more or less pointed.

The astragalar articular facets are relatively short, and the sustentacular facet (=distal articular facet) is comparatively slender and strongly curving. The ectal facet (=proximal articular facet) is oriented anterodorsally-posteroventrally, but nearly horizontal. Anteriorly, the sustentacular facet transits into the cuboid facet. The concave and lozenge-shaped cuboid facet is slightly higher than wide and overall small, restricted to the dorsal two thirds of the distal margin of the calcaneum.

**Navicular (Supplementary figure 2F, G).** One right (IRSNB 1187-M273f) and one left (IRSNB 1187-M273[no letter]) naviculars have been preserved along the two partially articulated and associated pes. Muizon [3] already pointed out that the navicular is of very limited use in the separation of different phocid species. Anteroposteriorly, the navicular is slightly compressed with a robust, but not particularly highly-raised ventral protuberance for the articulation with the astragalus. According to Muizon [3], *Acrophoca*, *Piscophoca*, and Phocinae have an articular facet for the entocuneiform that is sub circular, while this facet is rather oval in other phocids [3]. In this specimen, this articular facet for the entocuneiform is oval, as in Monachinae (except *Acrophoca* and *Piscophoca*). Although the flat facet for the entocuneiform is clearly outlined, this facet touches the meso- and ectocuneiform facets, which are faintly saddle-shaped (cuneiform facets in distal surface and not visible in Supplementary figure 2). At the medial margin of the navicular, the ectocuneiform facet makes a smooth 60° angle with the small triangular facet for the cuboid (not visible in Supplementary Figure 2).

**Cuboid (Supplementary Figure 2H-K).** One right cuboid (IRSNB 1187-M273e) is present. This cuboid articulates with the calcaneum and the navicular from the partially articulated pes (IRSNB 1187-M273f). As for the navicular, the cuboid is of little descriptive value and of little use to discern different phocid taxa. However, Muizon (1981) identified the presence of an articular facet for Mt V on the ventrolateral apophysis (= posterolateral apophysis in Muizon, 1981) as a phocine character, and its absence as a monachine character. This facet is difficult to see on cuboid IRSNB 1187-M273e, but the corresponding facet on the articulating Mt V (IRSNB 1187-M273j) is clearly visible and well-defined. In addition, this ventrolateral process is slender and surrounds a deep sulcus for the tendon of *m. peroneus longus*.

**Metatarsals (Supplementary Figure 3).** Five metatarsals are part of the partially articulated pes: right and left Mt I (IRSNB 1187-M273h,i), right Mt II (IRSNB 1187-M273g), left Mt IV (IRSNB 1187-M273k), and right Mt V (IRSNB 1187-M273j). The distal portion of Mt V is missing, precluding size comparison with other metatarsals. However, it appears that MtI and Mt V were the largest metatarsals, followed by Mt II and Mt IV. Despite the absence of Mt III in this pes, Mt III is by far the smallest metatarsal in Monachine, and only slightly smaller than Mt IV in Phocinae [5]. The proximal epiphysis of Mt I is much more expanded than the distal epiphysis. In proximal view the proximal epiphysis is trapezoidal in outline; it has a moderately concave facet for the articulation with the entocuneiform. This facet appears as a horizontally oriented hourglass in outline. The medial and dorsal edges of this facet are sharp. Laterally, this facet smoothly curves into an oval-shaped facet for the Mt II, which has an anterodorsal-posteroventral orientation. The ventral portion of the contact between the facet for the entocuneiform and the facet for Mt II projects proximally, forming a distinct process. On the dorsomedial margin of the proximal epiphysis of Mt I, there is a clearly delineated, concave ovoid muscle attachment surface. The shaft of Mt II is strongly medially offset to the proximal epiphysis. This is a characteristic that generally separates Monachinae from Phocinae; the latter having a Mt II that is less offset. The proximal epiphysis of Mt II bears a concave saddle-shaped facet for the articulation with the mesocuneiform. Muizon [3] noted that in Monachinae this facet is generally less concave than in Phocinae. However, the condition in IRSNB 1187-M273g approaches that of Phocinae. On the medial surface of the proximal epiphysis, there is a roughly flat surface for the articulation with Mt I. Dorsally on the lateral surface, there are two facets: a small, flat, oval facet for the ectocuneiform, proximally, and a larger, concave, sub circular facet for Mt III, distally. Noticeably shorter than Mt II, Mt IV has clearly delineated articular facets on its proximal epiphysis. The articular facets for Mt III consist of dorsal and plantar sub oval facets. Both facets are strongly convex and separated by a deep sulcus. Just distal to these facets, Mt IV exhibits two small concave areas, which further define the convex facets. Such articular facets have also been observed in Phocinae, while the articular facets are much less convex in Monachinae, in which the sulcus is much less pronounced. Proximally, the plantar facet of the Mt IV IRSNB 1187-M273k curves into the facet for the cuboid, as in Monachinae. This facet is elongate and oriented plantarmedially-dorsolatarally. On the lateral surface of the proximal epiphysis, the articular facet for Mt V is slender and curving, but not particularly elongate. Arching along the dorsoproximal margin of the bone, both extremities of this facet are slightly expanded and separated by a pit. Whereas Mt I is slightly compressed plantardorsally and Mt II and Mt IV are more rounded in cross-section, the shaft of Mt V is slightly compressed mediolaterally. Being very broad plantardorsally at its proximal epiphysis, the articular facet for Mt IV follows the proximodorsal margin of the bone cylindrically. Proximally, this articular facet rounds the dorsal margin to the medial surface of the bone, where it articulates with the cuboid, as in Phocinae. Proximoventrally on the proximal epiphysis, a cylindrical facet for the ventrolateral process of the cuboid is present, as in Phocinae.

**Phalanges (Supplementary figure 4).** For the partial right and left pes (IRSNB 1187-M273), multiple phalanges have been preserved. However, their exact positions within the pes remain unknown. The phalanges are dorsoventrally flattened and have relatively smooth distal epiphyses, which are typically lobodontin and monachine traits, respectively, while the phalanges of Phocinae are more rounded in cross-section and have prominently keeled distal epiphyses [3, 5]. Although there are more traits linking pes IRSNB 1187-M273 to Monachinae than to Phocinae, a number of phocine characters have been observed (see metatarsals). These phocine traits cannot be ignored, but smoothened phalangeal epiphyses are considered a ‘derived’ or synapomorphic monachine trait [6]. Therefore, we consider this pes to be Monachinae indet.

**Sexual dimorphism?** Given the overall relatively small size and the slender nature of the calcaneum IRSNB 1187-M273d, its size rather corresponds to that of the isolated lectotype humerus of *Frisiphoca aberratum* (IRSNB 1191-M266). The presence of a baculum in this specimen clearly shows that it represents a male, which precludes the possibility that the smaller isolated lectotype humerus of *F. aberratum* and the larger lectotype humerus of *Frisiphoca* *affine* represent sexual dimorphism within the one single species, with the former representing a female specimen and the latter representing a male specimen.

**Left pes IRSNB 1188-M270**

**Metatarsal (Supplementary figure 5A, B).** The left fifth metatarsal IRSNB 1188-M270b differs noticeably from the right fifth metatarsal of the more complete pes (IRSNB 1187-M273j). Specimen IRSNB 1188-M270b is more slender than IRSNB 1187-M273j and the proximal epiphysis is less elongate plantardorsally. Proximoventrally on the proximal epiphysis, a cylindrical facet for the ventrolateral process of the cuboid is present, as in Phocinae and IRSNB 1187-M273j. The presence of a pronounced pit for the insertion of *m. peroneus brevis* on the lateral side of the proximal epiphysis in IRSNB 1188-270b differs from the condition in IRSNB 1187-M273j, in which this pit is much less pronounced. The proximoventral margin of the bone is slightly convex, as in IRSNB 1187-M273j.

**Phalanx (Supplementary figure 5C, D).** The left phalanx IRSNB 1188-M270a does not differ noticeable from the phalanges of IRSNB 1187-M273. The phalanx is typically monachine: the diaphysis is plantardorsally flattened and the distal epiphysis is smoothened, not bearing a keel, as is the case in Phocinae. The shape of the phalanx leads to conclude that specimen IRSNB 1188-M270 is monachine, rather than phocine.

**Associated phalanges (Supplementary figure 6).** Four partial phalanges have formerly been assigned to *Monatherium affine*. However, given their state of preservation and the limited diagnostic value of phalanges, it is impossible to support this assignment. The dorsopalmar or dorsoplantar flattening of the diaphyses and the smoothening of the distal epiphyses are typically monachine characteristics. Therefore, it is safe to consider these isolated phalanges Monachinae indet.

**Isolated specimen**

**Calcaneum (Supplementary figure 7A-C).** One isolated right calcaneum (IRSNB M1144) has been assigned to *Monatherium aberratum*. Unlike the calcaneum of Phocinae, this calcaneum and calcanea IRSNB 1187-M276d (originally *M. aberratum*; Monachinae indet. in this study) and IRSNB 1125-M263 (originally *Monatherium affine*; Phocidae *cf. Frisiphoca affine* in this study) are distally much wider than they are proximally, in lateral view. The ratio of the plantardorsal height to the total length of the calcaneum is 0.485 (Supplemental information 1; Supplemental table 4), which lies just within the range of Phocinae, based on Muizon [3: table 8]. Extant Lobodontini have a high ratio (>0.55), other extinct phocids and extant Monachini an intermediate ratio, and extant phocines a low ratio (<0.50). Because of the unclear distinction of many extinct phocid calcanea from both subfamilies based on this ratio, this value of 0.485 is of little diagnostic value. The calcaneal tuber does not bear a prominent medial process (=medial calcaneal tuber) at its proximal end, contrasting to IRSNB 1125-M263 and IRSNB 1187-M276d. On the dorsal margin of the calcaneum, approximately at one-third of the length of the calcaneum, there is an oval concave facet for the articulation with the fibula. Among Phocidae, only extant Lobodontini and *Homiphoca* have a well-developed fibular facet on the calcaneum.

Forming a longitudinal crest, the trochlear process extends across the dorsal surface of the calcaneum anterior to the ectal facet (=proximal astragalar facet), as in other Phocidae [4]. On the lateral margin of the calcaneum, the peroneal process, supporting the tendon of *m. peroneus longus* and *m. peroneus brevis*, is well developed, as in IRSNB 1187-M276d, which is a monachine trait [3]. The sustentaculum is wider than in IRSNB 1187-M276d, and the secondary shelf of the sustentaculum is less prominent and much more rounded than in the latter.

The astragalar articular facets are relatively short, and the sustentacular facet (=distal articular facet) is wide and almost straight with sharply curving extremities. The ectal facet (=proximal articular facet) is oriented anterodorsally-posteroventrally. Anteriorly, the sustentacular facet forms a sharp angle with the cuboid facet.

Overall, calcaneum IRSNB M1144 does not differ significantly from IRSNB 1187-M276d. Although the elongated size of the specimen points towards an indentification as a phocine [3: table 8], this remains questionable in the absence of many measurements of other extinct phocine calcanea. Other observations generally favor assigning IRSNB M1144 to Monachinae.

**Subfamily Phocinae indet.**

**Referred specimens. *Associated hind limb elements*.** IRSNB 1189-M271a-b: left second and third metatarsal (a), distal part of left fibula (b), and left ectocuneiform (?), 'Diestian', 3^rd^ section at Deurne, Antwerp, Belgium, originally assigned to *Monatherium aberratum* by Van Beneden [1: plate 17, figures 12, 13]. ***Isolated radius.*** IRSNB 1138-M267, left radius, 'Diestian', 3^rd^ section at Borgerhout, originally assigned to *Monatherium aberratum* by Van Beneden [1: plate 17, figure 5]. ***Isolated tibia*.** IRSNB 1122-M264, left tibia, 'Diestian', 3^rd^ section, ?Borgerhout, originally assigned to *Monatherium affine* by Van Beneden [1: plate 16, figure 14]. ***Isolated phalanges.*** IRSNB 1217-M256b, unspecified phalanx, ‘Diestian’, 3^rd^ section at Borgerhout, originally originally assigned to *Monatherium delognii* by Van Beneden [1: plate 16, figure 4]; IRSNB M1227, two unspecified phalanges, originally assigned to *Monatherium delognii* [1].

**Comments.** A detailed reassessment of the original ‘Diestian’ attribution of the original specimens of *Monatherium* from Belgium is provided in the main text and not in the supplementary information.

**Hind limb IRSNB 1189-M271**

**Fibula (Supplementary figure 8A, B).** The distal part of the left fibula IRSNB 1189-M271b is preserved; it is very slender. As in other Phocidae, a ridge separates the tendon grooves of *m. peroneus brevis* and *m. extensor digitorum lateralis*. This ridge terminates in a circular protuberance that deflects posteriorly.

**Ectocuneiform (Supplementary figure 8C, D).** The left ectocuneiform of IRSNB 1189-M271 is small and phocine in shape. Muizon [3] showed that the ratio of the anteroposterior height of the ectocuneiform to the plantardorsal length of the bone is higher in Phocinae than in Monachinae. With a value of 0.699 (13.0 mm : 18.6 mm), this ratio approaches the values of Phocinae (0.7) from Muizon [3: table 7] (approximately 0.55-0.66 for Monachinae). The reduction of the plantar process has also been observed in Phocinae.

**Metatarsals (Supplementary figure 8E-J).** The second and third left metatarsals (both IRSNB 1189-M271a) are present. As with the fifth metatarsals IRSNB 1187-M273j versus IRSNB 1188-M270b, Mt II IRSNB 1189-M271a is much more slender than IRSNB 1187-M273g (Monachinae indet., see above). The shaft of the bone is less offset here than it is in IRSNB 1187-M273g, associating the two metatarsals of IRSNB 1189-M271a with Phocinae, rather than with Monachinae. The proximal articular surface for the mesocuneiform is more strongly concave than it is in IRSNB 1187-M273g and the articular facet for the entocuneiform on the medial margin of the proximal epiphysis is deeper as well. Mt III is noticeably shorter than Mt II and has a proximal epiphysis that is typically triangular in proximal view. Dorsal on the medial and lateral sides of the proximal epiphysis, there are a slightly convex articular surface for Mt II and a slightly concave articular surface for Mt IV, respectively. Ventrally on the proximal surface of the proximal epiphysis, there are two small and vertically oriented facets that face approximately 90° apart. The medial facet articulates with the ectocuneiform and the lateral facet with the cuboid.

**Isolated specimens**

**Radius IRSNB 1138-M267 (Supplementary figure 9A, B).** The isolated left radius IRSNB 1138-M267 was originally assigned to *Monatherium aberratum* [1]. Only the proximal part of this radius is preserved, but it is skeletally mature [2]. Its size conforms the size of the lectotype humerus of *Frisiphoca aberratum* IRSNB 1191-M266, and is much too small for the lectotype humerus of *Frisiphoca affine* IRSNB 1118-M260.

The articular facet for the ulna is well outlined, with a prominent medial tip. The articular facet for the humerus strongly overhangs the diaphysis anterolaterally. The bicipital tuberosity is located on the posteromedial surface of the diaphysis, which matches the condition in extant Monachinae, but differs from currently known extinct Monachinae, in which the bicipital tuberosity is located more medially. Among Phocinae, the location of this tuberosity varies interspecifically. The bicipital tuberosity is little pronounced, circular in shape, and does not contact the articular facet for the ulna. A lowly raised but distinct ridge on the lateral surface of the diaphysis points towards a strongly developed *m. supinator*. Overall, the preserved part of the diaphysis indicates that the diaphysis is comparatively thin, as in Phocinae, but contrasting to Monachinae.

Nevertheless, because this radius is poorly preserved, and because it was found isolated, its attribution to *F. aberratum* is not well supported. It is more appropriate to degrade this specimen to Phocinae indet.

**Tibia (Supplementary figure 10).** The partial tibia IRSNB 1122-M264 lacks its distal portion. Even though it is incomplete, the specimen corresponds to a large seal, comparable in size to the extant *Leptonychotes weddelli*. The tibial plateau bears a small medial articular condyle for the femur, and a large lateral condyle. Both condyles are slightly concave. The anterior margin of the lateral condyle forms a sharp edge. The intercondyloid area is divided in a concave anterior area and a posteriorly sloping posterior intercondyloid area by a raised intercondyloid eminence. The tibial plateau is strongly similar to that of the early-branching stem phocine *Prophoca rousseaui* [7], but the phocid tibia is of limited diagnostic value [8]. The patellar facet on the anterior margin of the tibia is semi lunate in outline. Due to the incompleteness of the tibia, the degree of curvature of the bone is difficult to elucidate. Both pre-tibial and post-tibial fossae are well developed, which places IRSNB 1122-M264 closer to phocines than to monachines, despite its overall incompleteness.

**Phalanges (Supplementary figure 11).** Two phalanges in this study (IRSNB 1217-M256b and IRSNB M1227) were originally identified as *Monatherium delognii* and are reassigned here to Phocinae indet. All three phalanges are only partially preserved. For IRSNB 1217-M256b, only the diaphyseal part of the phalanx is preserved, and for both specimens IRSNB 1227, only the distal half of the phalanx is preserved. In cross-section, the diaphysis is relatively sub circular. The preserved distal epiphyses are strongly keeled. These characteristics suggest phocine affinities, while Monachinae generally have phalanges that are flattened in cross-section and with smoothened distal epiphyses [3, 6]. Especially the strongly keeled epiphyses are considered a primitive trait retained in Phocinae and other non-phocid pinnipeds, while the rounded epiphyses in Monachinae are considered to be a derived trait [6].

**Family Phocidae indet.**

**Referred specimens. *Associated vertebrae.*** IRSNB 1108-M255a,b, thoracic vertebra (a), lumbar vertebra (b), 'Diestian', 2^nd^ section, Borgerhout, Antwerp, Belgium, originally assigned to *Monatherium delognii* by Van Beneden [1: plate 16, figures 1, 2]. IRSNB 1132-M269a,b, thoracic vertebra (a), cervical vertebra (b), ‘Diestian’, 3^rd^ section at Borgerhout, originally assigned to *Monatherium aberratum* by Van Beneden [1: plate 17, figures 7-9]. ***Associated lumbar vertebra, innominate, and pes.*** IRSNB M1219, lumbar vertebra, left innominate, left MtIV, and eight phalanges, originally assigned to *Monatherium aberratum* [1]. ***Associated humerus and innominate.*** IRSNB M1218, partial left humerus, partial left innominate, originally assigned to *Monatherium affine* [1]. ***Isolated lumbar vertebrae.*** IRSNB M1114, lumbar vertebra, originally assigned to *Monatherium affine* [1]*.* IRSNB M1190, lumbar vertebra, originally assigned to *Monatherium affine* [1]. ***Isolated sacrum.*** IRSNB M1216, originally assigned to *Monatherium delognii* [1]. ***Isolated caudal vertebrae.*** IRSNB 1217-M256a, third? caudal vertebra, ‘Diestian’, 3^rd^ section at Borgerhout, originally assigned to *Monatherium delognii* [1: plate 16, figure 3]; IRSNB M1217, fifth? caudal vertebra, originally assigned to *Monatherium delognii* [1]. ***Isolated sternebrum.*** IRSNB 1219-M268, sternebrum, ‘Diestian’, 3^rd^ section, Borgerhout, originally assigned to *Monatherium aberratum* by Van Beneden [1: plate 17, figure 6]. ***Isolated radii.*** IRSNB 1121-M261b, left radius originally proposed to be associated with ulna IRSNB 1121-M261a, but markedly different in size, 'Diestian', Borgerhout, originally assigned to *Monatherium affine* by Van Beneden [1: plate 16, figure 11]; IRSNB M1139, right radius, originally assigned to *Monatherium delognii* [1]. ***Isolated fibula.*** IRSNB M1149, left? fibula, originally assigned to *Monatherium delognii* [1]. ***Isolated astragalus.*** IRSNB 1144-M272, right astragalus, ‘Diestian’, 3^rd^ section at ?Borgerhout, originally assigned to *Monatherium aberratum* by Van Beneden [1: plate 17, figure 14].

**Comments.** Part of specimens originally attributed to the three *Monatherium* species from Belgium are unidentifiable beyond the family level and are considered Phocidae indet. Biostratigraphic analysis of a dinoflagellate cyst assemblage associated with either specimen IRSNB 1108 or with specimen IRSNB 1108-M255 (formerly *M. delognii*) yields a maximum age of 13.2 Ma, but no practical minimum age limit for the sediment sample. A detailed reassessment of the original ‘Diestian’ attribution of the original specimens of *Monatherium* from Belgium is provided in the main text and not in the supplementary information.

**Description and comparison**

**Associated vertebrae**

**Thoracic and lumbar vertebrae (IRSNB 1108-M255a, b) (Supplementary figure 1D-I)**. Two very large vertebrae have originally been assigned to *Monatherium delognii*: one thoracic vertebra (IRSNB 1108-M255a) and one lumbar vertebra (IRSNB 1108-M255b). The size of these vertebrae exceeds that of all other Phocidae, except the extant *Mirounga*. Both vertebrae are much longer than they are wide. However, both are only very partially preserved, lacking the neural arches and most of the transverse processes. Consequently, it is impossible to diagnose the preserved vertebrae to either Monachinae or Phocinae.

**Cervical and thoracic vertebrae (IRSNB 1132-M269a, b) (Supplementary figure J-O)**. Both vertebrae are very incompletely preserved, missing significant portions of their neural arches and transverse processes. The vertebral body of the cervical vertebra (IRSNB 1132-M269b) is much longer than the body of the thoracic vertebra (IRSNB 1132-M269a). This contrasts with the general phocid axial skeleton, in which the bodies of the cervical vertebrae are much smaller than the bodies of the thoracic and lumbar vertebrae [9]. Therefore, the association of the two vertebrae IRSNB 1132-M269a and b to a single individual can be questioned.

The cervical vertebra is relatively large, clearly belonging to a large seal, comparable in size to the extant monachines *Hydrurga leptonyx* and *Leptonychotes weddelli*. The body of this vertebra is strongly elongate and bears a prominent ventral crest, which becomes more pronounced posteriorly, giving the body a contracted appearance. The anterior and posterior articular facets of the body are tilted forward.

The smaller body of the thoracic vertebra has vertical anterior and posterior articular facets. It lacks a central ventral crest. Two concave articular facets for the ribs are present, the anterior and the posterior costal fovea. The anterior costal fovea is located at the anterodorsal margin of the lateral surface of the body and facing anteroventrolaterally. The posterior costal fovea is located on the dorsal half of the posterior surface of the vertebral body and faces posteriorly. Despite these descriptive elements, phocid vertebrae bear little diagnostic characteristics and it is impossible to identify IRSNB 1132-M269a and b to the subfamilial level.

**Associated lumbar vertebra, innominate, and pes (IRSNB M1219)**

**Lumbar vertebra (Supplementary figure 1P-R).** The lumbar vertebra is usually of limited diagnostic value [3]. Lumbar vertebra IRSNB M1219 is moderately well preserved, but misses all processes, rendering it even less valuable for diagnosis. The vertebra belongs to a medium-sized phocid, comparable in size to *Erignathus barbatus*. The posterior articular surface is preserved and is subcircular. The neural arch is strongly dorsoventrally flattened, yielding a kidney-shaped neural canal in anterior view.

**Innominate (Supplementary figure 12A, B).** Although the innominate is much more useful than many other postcranial remains in distinguishing between Monachinae and Phocinae [3, 10], the partial left innominate M 1219 is too incompletely preserved. Only the anterior half of the acetabulum and the ventral part of the ilium are preserved. Due to this incomplete preservation, it is impossible to assess the degree of lateral eversion of the iliac wing in relation to the rest of the bone, which is an important character used to differentiate Phocinae (strong eversion) and Monachinae (weak eversion), although a number of extinct Phocinae also show a relatively weak lateral eversion of the ilium (e.g., *Nanophoca vitulinoides* and *Prophoca rousseaui*; [7, 11]). However, it is clear that the gluteal fossa on the lateral surface of the ilium is only weakly developed. In other Phocidae, a weakly developed gluteal fossa is present among Monachinae and some extinct Phocinae, such as *Prophoca rousseaui* [11]. Due to this overlap in extinct Monachinae and Phocinae, it is impossible to assign innominate IRSNB M1219 to either of both subfamilies.

**MtIV (Supplementary figure 12C-E).** The articular facets for Mt III consist of dorsal and plantar sub oval facets, a feature shared with Phocinae and the extinct monachines *Acrophoca longirostris* and *Piscophoca pacifica* [3]. Both facets for MtIII on the M1219 MtIV are strongly convex and separated by a deep sulcus. The two small concave areas just distal to these facets, which are present in IRSNB 1187-M276k (Monachinae indet., see above), are also present in specimen IRSNB M1219, but less developed. Just distal to these facets, Mt IV exhibits two small concave areas, which further define the convex facets. Such articular facets have also been observed in Phocinae, while the articular facets are much less convex in Monachinae. The articular facet for Mt V is rather short, as in Monachinae.

**Phalanges (Supplementary figure 12F-U).** Eight phalanges are present in specimen IRSNB M1219. The state of preservation varies between the different phalanges. One phalanx has its distal and proximal epiphyses preserved, one phalanx its proximal epiphysis, and three phalanges their distal epiphysis. The distal epiphysis is generally strongly rounded and keeled, which is considered a phocine character, while the distal epiphysis of the phalanx is usually relatively flat and smooth in Monachinae.

Consequently, these phalanges can be regarded as being phocine. However, the associated lumbar vertebra, innominate, and mtIV bear a number of monachine characters. Thus, given the overall poor state of preservation and the limited diagnostic value of the specimens, it is more appropriate to treat specimen IRSNB M1219 as Phocidae indet.

**Associated humerus and innominate.**

**Humerus (Supplementary figure 13A).** Specimen IRSNB M1218 includes one very partially preserved distal extremity of a left humerus. The lateral epicondylar crest is moderately well preserved. Among modern phocids, this crest is only present in Phocinae. However, it is also present in *Frisiphoca affine* (see main text), which has unknown subfamilial relationships. Overall, an identification as Phocinae would be only very tentative and, hence, we consider this specimen Phocidae indet.

**Innominate (Supplementary figure 13B, C).** The partial left innominate IRSNB M1218 is only partially preserved. It is large, corresponding in size to that of the extant monachine *Leptonychotes weddelli*. The lateral eversion of the ilium is only moderately well developed and the development of the gluteal fossa on the lateral side of the ilium is weak. These characteristics are shared with Monachinae and a number of extant and extinct Phocinae, including *Erignathus barbatus* and *Prophoca rousseaui* [7]. Hence, it is impossible to identify innominate IRSNB M1218 beyond the family level.

**Isolated specimens**

**Lumbar vertebra (IRSNB M1114) (Supplementary figure S-U).** Specimen IRSNB M1114 is an isolated lumbar vertebra, which had formerly been assigned to *Monatherium affine* [1]. However, only a portion of the vertebral body is preserved, rendering it only possible to assign it to the Phocidae family. The size of the vertebra is comparable to that of a large seal, such as *Hydrurga leptonyx.*

**Lumbar vertebra (IRSNB M1190) (Supplementary figure V-X).** This isolated lumbar vertebra was also formerly assigned to *Monatherium affine* [1]. Similar to IRSNB M1114, the size of the vertebra is comparable to that of a large phocid, e.g., *Leptonychotes weddelli*. The neural arch is partially preserved, showing a dorsoventrally flattened rectangular neural canal in anterior view. The anterior prezygapophyses are approximately at an angle of 90° from each other. Due to the incompleteness of the specimen, combined with the overall limited diagnostic value of phocid vertebrae, it is impossible to identify this specimen beyond the family level.

**Sacrum (Supplemental figure 14).** IRNSB M1216 was the second sacrum assigned to *Monatherium delognii* by Van Beneden [1], apart from the specimen IRSNB 1153-M257a, which has been selected as the lectotype (see main text). Specimen IRSNB M1216 differs in having a strong ventral deflection of the ventral margins of the sacral wings, while horizontally straight ventral margins of the sacral wings are characteristic for *Prophoca rousseaui*. However, given the overall incomplete state of preservation of the specimen, the absence of the sacrum in the known fossil record of many Monachinae and Phocinae from the North Atlantic, and the difficult distinction between the sacra of Monachinae and Phocinae [7], it is impossible to assign specimen IRSNB M1216 to either Monachinae or Phocinae. It is hence regarded as Phocidae indet.

**Caudal vertebrae (Supplementary figure 1Y-D’).** IRSNB 1217-M256a and IRSNB M1217 are caudal vertebrae of comparable size and shape, and both had originally been assigned to *Monatherium delognii* [1]. IRSNB 1217-M256a had originally been associated with a partial phalanx (IRSNB 1217-M256b), but the association of a caudal vertebra and a phalanx without other associated bones remains questionable. Both specimens are large, comparable in size to the extant monachine *Leptonychotes weddelli.* The caudal vertebrae have strongly anteroposteriorly elongate transverse processes, which are slightly upturned. The specimens lack a completely closed neural arch, but still bear their prezygapophyses with associated mammillary processes. The prezygapophyses lack articular facets. These caudal processes do not differ from caudal processes in other Phocidae and it is very difficult to elucidate whether these individual vertebrae are monachine or phocine in the absence of a more completely preserved series of caudal vertebrae [3].

**Sternebrum.** Only one isolated sternebrum (IRSNB 1219-M268) is present in the entire collection of fossil Phocidae at the RBINS. This sternebrum is roughly rectangular cuboid shaped, with rounded lateral margins. Unfortunately, the sternebrum does not bear any diagnostic value to separate Monachinae from Phocinae.

**Radius (IRSNB 1121-M261b) (Supplementary figure 9C-D).** The left radius IRSNB 1121-M261b has been found associated with the right ulna IRSNB 1121-M261a (Phocidae *cf. Frisiphoca* in this study) in the RBINS collection. However, both seem to represent different stages in skeletal maturity. While the preserved portion of the ulna indicates a sub adult to adult specimen, the radius misses its proximal and distal epiphyses, indicating a skeletally juvenile specimen (for extant Phocinae see Storå, [2]). The radius differs strongly from the other isolated radius (IRSNB 1138-M267, identified as Phocinae indet. in the current study). The incompleteness of both specimens and the juvenile nature of IRSNB 1121-M261b preclude a detailed comparison and description. In adult Phocidae, the radii of Monachinae can be easily distinguished from the radii of Phocinae in displaying a diaphysis that widens more strongly distally. However, this trait is less conspicuous in juvenile radii, rendering any subfamilial designation of IRSNB 1121-M261b difficult.

**Radius (IRSNB M1139) (Supplementary figure 9E-G).** Another radius is only known from its proximal portion. It is impossible to assign a radius to either Monachinae or Phocinae, based on its proximal epiphysis alone. The bicipital tuberosity is located posteromedially, which corresponds to extant Lobodontini and *Monachus* [3]. However, this condition is variable in Phocinae, and, hence, cannot be used as a characteristic separating both subfamilies.

**Fibula (Supplementary figure 15).** One isolated partial fibula had originally been assigned to *Monatherium delognii* [1]. The most diagnostic pelvis of *M. delognii* (IRSNB 1153-M276) shows affinities with the phocine *Prophoca rousseaui* (this study). Given the current absence of a fibula in the fossil record of *P. rousseaui*, the identification of fibula IRSNB M1149 as *M. delognii* is rendered void. Additionally, this fibula is so poorly preserved that it cannot be described adequately and it cannot be specified beyond the family level: Phocidae indet.

**Astragalus (Supplementary figure 16).** One partial right astragalus (IRSNB 1144-M272) had been found isolated. However, it matches the articulated calcaneum IRSNB 1187-M273d (identified as Monachinae indet. in the present study) in size. The tibial facet is small, with both lateral and medial facets approximately at a 90° angle. The tip of the lateral tibial facet is well developed, strongly protruding, and slightly recurving dorsally, which gives the lateral tibial facet a faintly concave appearance. In lateral view, the tibial facet is convex. The caudal process is strongly developed, as in other Phocidae, and it is mediolaterally thick. The head of the astragalus is severely damaged, making the description of the shape of the sustentacular facet impossible. The ectal facet (=proximal calcaneal facet) is anteroposteriorly elongate, but overall relatively short, as in *Monachus*, *Piscophoca*, *Pliophoca*, and Phocinae. The medial interarticular sulcus, separating the ectal facet from the missing sustentacular facet, is moderately deep. Considering the generally poor state of preservation and the occurrence of characters that are shared with Phocinae and (some) Monachinae, it is impossible to identify astragalus IRSNB 1144-M272 beyond the family level.

**References**

1. Van Beneden P-J. 1877 Description des ossements fossiles des environs d’Anvers, première partie. Pinnipèdes ou amphithériens. *Annales du Musée Royal d’Histoire Naturelle de Belgique* 1, 1–88.
2. Storå J. 2000 Skeletal development in the Grey seal *Halichoerusu grypus*, the Ringed seal *Phoca hispida bontica*, the Harbour seal *Phoca vitulina vitulina* and the Harp seal *Phoca groenlandica*. Epiphyseal Fusion and Life History. *Archaeozoologia* 11, 199–222.
3. Muizon C de. 1981 Les vertébrés fossils de la Formation Pisco (Pérou) Première partie: deux nouveaux Monachinae (Phocidae: Mammalia) du Pliocène de Sud Sacaco. *Institut Français d’Etudes Andines, Mémoire* 6, 20–161.
4. Robinette HR, Stains HJ. 1970. Comparative study of the calcanea of the Pinnipedia. *Journal of Mammalogy* 51, 527-541.
5. Wyss AR. 1988 On “retrogression” in the evolution of the Phocinae and phylogenetic affinities of the Monk seals. *America Museum Novitates* 2924, 1-38.
6. Berta A, Wyss AR. 1994 Pinniped phylogeny. *Proceedings of the San Diego Society of Natural History* 29, 33-56.
7. Dewaele L, Lambert O, Louwye S. 2017 On *Prophoca* and *Leptophoca* (Pinnipedia, Phocidae) from the Miocene of the North Atlantic realm: redescription, phylogenetic affinities and paleobiogeographic implications. *PeerJ* 5*,* e3024. (doi:10.7717/peerj.3024)
8. Koretsky IA. 2001 Morphology and systematics of the Miocene Phocinae (Mammalia: Carnivora) from Paratethys and the North Atlantic Region. *Geologica Hungarica series Palaeontologica* 54, 1–109.
9. Berta A, Kienle S, Bianucci G, Sorbi S. 2015 A Reevaluation of *Pliophoca etrusca* (Pinnipedia, Phocidae) from the Pliocene of Italy: phylogenetic and biogeographic implications. *Journal of Vertebrate Paleontology* 35, e889144. (doi:10.1080/02724634.2014.889144)
10. Bininda-Emonds ORP, Russell AP. 1996 A morphological perspective on the phylogenetic relationships of the extant phocid seals (Mammalia: Carnivora: Phocidae). *Bonner Zoologische Monographien* 41, 1-256.
11. Dewaele L, Amson E, Lambert O, Louwye S. 2017 Reappraisal of the extinct seal “*Phoca*” *vitulinoides* from the Neogene of the North Sea Basin, with bearings on its geological age, phylogenetic affinities, and locomotion. *PeerJ* 5, e3316. (doi:10.7717/peerj.3316)
